# Taxonomic Revision of the Catostemma Clade (Malvaceae/Bombacoideae/Adansonieae)

**DOI:** 10.3390/plants14142085

**Published:** 2025-07-08

**Authors:** Carlos Daniel Miranda Ferreira, William Surprison Alverson, José Fernando A. Baumgratz, Massimo G. Bovini

**Affiliations:** 1 Instituto de Pesquisas Jardim Botânico do Rio de Janeiro, Diretoria de Pesquisas, Rua Pacheco Leão, 915, Rio de Janeiro CEP 22.460-030, Brazil; jbaumgra@gmail.com (J.F.A.B.); bovinimassimo@gmail.com (M.G.B.); 2 Wisconsin State Herbarium, University of Wisconsin-Madison, Birge Hall, 430 Lincoln Drive, Madison, WI 53706, USA; quararibea@gmail.com

**Keywords:** Amazon, *Aguiaria*, *Catostemma*, conservation status, neotropical trees, *Scleronema*

## Abstract

The Catostemma clade comprises three genera: *Aguiaria*, *Catostemma*, and *Scleronema*. These genera are representatives of the tribe Adansonieae, and are part of the subfamily Bombacoideae of the Malvaceae family. Taxonomic studies of these genera are scarce and limited to isolated publications of new species or regional floras. We reviewed their taxonomy, morphology, and geography, and assessed gaps in our knowledge of this group. We carried out a bibliographic survey, an analysis of herbarium collections, and collected new material in Brazilian forests. Here, we provide an identification key, nomenclatural revisions, morphological descriptions, taxonomic comments, geographic distribution maps, illustrations, and analyses of the conservation status for all species. We also discuss probable synapomorphies of the clade, to advance our understanding of phylogenetic relationships within the Adansonieae tribe of Bombacoideae. In total, we recognize 16 species: 1 *Aguiaria*, 12 *Catostemma*, and 3 *Scleronema*, of which 7 are endemic to Brazil, 1 to Colombia, and 1 to Venezuela. Two species are ranked as Critically Endangered (CR), and four as Data Deficient (DD).

## 1. Introduction

The Catostemma clade is represented by three genera, *Aguiaria* Ducke, *Catostemma* Benth., and *Scleronema* Benth. Phylogenetic work places the genera in the tribe Adansonieae, subfamily Bombacoideae, of the family Malvaceae [1]. All species are trees, 10–50 m tall, with unifoliate adult leaves (or digitately compound adult leaves in *Catostemma digitatum* J.D.Sheph. & W.S.Alverson, and sometimes in *Catostemma commune* Sandwith), bearing flowers with lobed calyces, white to slightly pinkish or reddish corollas, stamens partially fused at the base into a tube, 2–5-carpellate ovaries with 2–7 ovules per locule, and fruits that usually are 1-seeded, either partially dehiscent capsules (*Aguiaria* and *Catostemma*), or indehiscent woody berries (*Scleronema*). The Catostemma clade is exclusively found in Amazonian forests, but also known from inter-Andean watersheds in Colombia (i.e., *C. digitatum*).

Previous studies of these genera are scarce [2,3,4,5,6,7], limited to occasional publications of new species and few monographic works for restricted regions, but never comprehensive. To address significant gaps in our knowledge, we analyzed available literature, searched herbarium collections, and conducted new fieldwork in Amazonian Brazil.

Initially thought to comprise 22 species, as a result of our analyses the clade is now represented by 16 (1 *Aguiaria*, 12 *Catostemma*, and 3 *Scleronema*), the reduction a result of the new synonyms proposed here, despite the recent addition of a new species. Furthermore, we recognize *Catostemma lemense* Sanoja as an uncertain species. Our review provides a key to the genera and species, morphological descriptions, taxonomic comments, distribution maps, illustrations, and analyses of conservation status. Additionally, we recognize probable synapomorphies for the Catostemma clade in hope that this will inform future studies of phylogenetic relationships within Adansonieae.

## 2. Materials and Methods

The present study involved a bibliographic search and analyses of herbarium specimens, including those of AMAZ, BM, BR, COAH, COL, CUZ, EAFM, F, FMB, HUA, HUAM, IAN, INPA, JAUM, K, L, MBM, MEDEL, MG, MO, MPU, NY, P, QCNE, RB, S, SP, U, UB, UFMT, US, VEN, W, and WIS [8], from which we obtained geographic distributions, morphological, and phenological data. Field expeditions were conducted in the Brazilian states of Acre, Amazonas, Pará, and Roraima to improve data on distribution, morphology, and habitat. Newly collected specimens were deposited in the herbarium of the Rio de Janeiro Botanical Garden Research Institute (RB). Because seedling leaf morphology is recognizably distinct from that of adult individuals, whenever material was available our descriptions are based on individuals analyzed in both the field and the herbarium.

Morphological characters were described following Beentje [9] (general morphology), Payne [10], and Theobald et al. [11] (for trichomes), and Ellis et al. [12] (for tertiary vein patterns). For descriptions of trichomes, leaf samples removed from specimens at the RB herbarium of *Aguiaria excelsa* Ducke (*Ferreira et al. 845* and *846*), *Catostemma lanceolatum* C.D.M.Ferreira & W.S.Alverson (*Demarchi & Barcelos 872*), *Scleronema spruceanum* Benth. (*Ferreira et al. 837* and *838*), and *S. praecox* (Ducke) Ducke (*Souza et al. 1933*) were used for SEM analysis with an Hitachi TM4000Plus at 15 kV. Our descriptions of leaf venation focused on secondary and tertiary veins in rectangular sections of leaflets removed from herbarium specimens, *Catostemma cavalcantei* Paula (*Rosa & Cordeiro 1690*, MG 53960) and *Scleronema grandiflorum* Huber (*Ferreira & Viana 870*, RB 834673). Leaf clearing and construction of the descriptions follows Ferreira et al. [13].

Species distribution maps were prepared with the ArcGIS Desktop program [14], using localities based on coordinates of herbarium specimens or recorded during fieldwork. Common names were obtained from specimen labels and from discussions with local residents during our fieldwork. IUCN guidelines [15] were used to assess conservation status. In our lists of specimens examined, we note phenology as flowering (fl.), fruiting (fr.), juvenile/seedling (seed.), or adult/sterile (st.). When the number of specimens analyzed for a species was very high, we listed additional specimens in Appendix A.

## 3. Results and Discussion

### 3.1. The Catostemma Clade

The Catostemma clade includes the *Aguiaria*, *Catostemma*, and *Scleronema* genera, with a combined total of 16 species. *Aguiaria* is distinguished from the other genera by the presence of flying buttresses on the trunks of adults, branch indumentum with dentate-lepidote trichomes, staminal columns with fasciculate trichomes (vs. glabrous), and fruit capsules with 5 valves (vs. capsules with 2–4 valves or indehiscent, woody berries). *Catostemma* differs from *Scleronema* by its staminal columns, which are wider than long or with similar length and width, excepting *C. commune* with columns rarely longer than wide (vs. staminal column longer than wide); 50–500 slender staminal filaments (vs. 12–30 thick filaments); pyriform ovaries (vs. globose to oblate or ovoid), and partially or tardily dehiscent fruits (vs. indehiscent woody berries). The Catostemma clade differs from other genera of tribe Adansonieae (*Adansonia* L. and *Cavanillesia* Ruiz & Pav.) by the absence of swollen trunks and by perigynous flowers (Figures 3F and 4O) (vs. swollen trunks, and hypogynous flowers). These data complement the phylogeny of Carvalho-Sobrinho et al. [1], suggesting the absence of swollen trunks and perigynous flowers as probable synapomorphies for the Catostemma clade.

### 3.2. Gross Leaf Morphology

Adult leaves of most species in the clade are unifoliolate compound, with the exception of two species of *Catostemma* that can be palmately 3-foliolate (*C. commune*) or are always 3-7-foliolate (*C. digitatum*). The unifoliolate-compound leaves provide no clear indication that they are not simple leaves, but we infer their compound nature for three reasons. First, their homology appears to be to the individual leaflets of the compound adult leaves of two species of *Catostemma* (noted above) and likewise to individual leaflets of compound-leaved seedlings of some species of *Catostemma*. Second, the Adansonieae tribe comprises the three genera of the Catostemma clade, plus *Adansonia* (with palmately compound leaves) and *Cavanillesia* (with simple leaves that have conspicuous palmate venation, suggesting homology to five or more, developmentally fused leaflets). Finally, the ancestral state of the Adansonieae tribe is likely palmately compound leaves, given that two of the three genera (*Bernoullia* Oliv. and *Gyranthera* Pittier) in the sister group, the Bernoullieae tribe, are palmately compound and the third genus (*Huberodendron* Ducke) appears to be unifoliolate-compound.

### 3.3. Trichome Morphology

We observed seven types of trichomes: stellate-rotate (Figure 1A), dentate-lepidote (Figure 1B), fasciculate (Figure 1C), simple (Figure 1D), glandular (Figure 1E), bifurcate, and dendritic. They are present in both vegetative and reproductive structures. Branches are glabrescent, with observable trichomes only on their apical regions. While some types of trichomes are common in most species such as the fasciculate, which make up the indumentum of ovaries and fruits of all species in the clade, others have great diagnostic relevance, occurring only in specific structures of a unique species. This is the case of the dentate-lepidote trichomes, which occur only in *A. excelsa*, on vegetative structures, pedicels, bracteoles, and calyces. Bifurcate trichomes also occur in only on the stipules of one species, *C. digitatum*. The absence and presence of trichomes are also of great importance in species recognition, as in the case of *Catostemma fragrans* Benth. It has fasciculate trichomes on the adaxial surface of the hypanthia and on the staminal filaments, unique to this species. A similar case occurs in *A. excelsa*, the only species bearing fasciculate trichomes on the staminal tube. The morphology and the presence or absence of trichomes proved to be important for the recognition of some of the species studied; however, in most cases it is necessary to observe other characters to reach a reliable identification.

Another important characteristic for recognition of some species is the presence of densely arranged stomata on the abaxial surface of the leaves (Figure 1F). These stomata may resemble glandular trichomes (Figure 1E); however, those structures are slightly elevated from the surface of the epidermis, whereas the stomata are slightly sunken when observed under a microscope.

### 3.4. Leaf Tertiary-Vein Morphology

**Figure 2:**
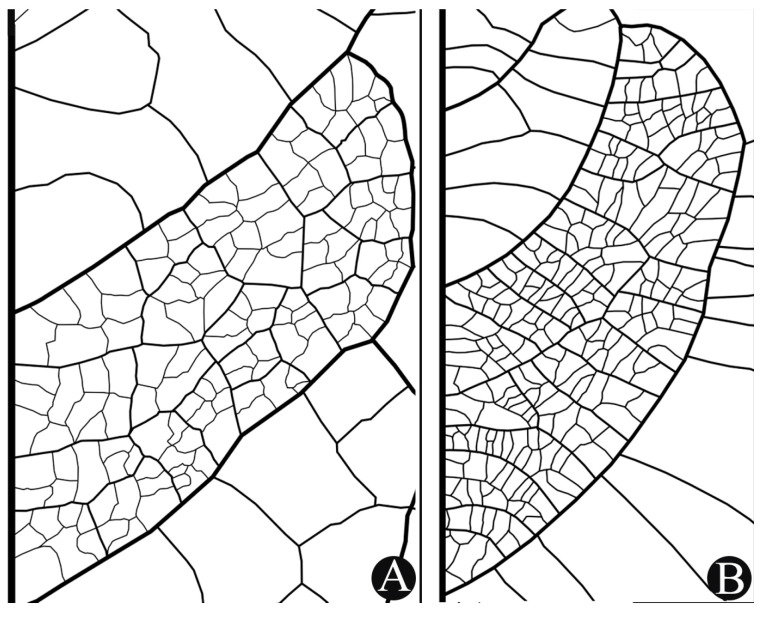
Tertiary veins on leaflets of the Catostemma clade. (**A**) Tertiary veins alternate percurrent, *Catostemma cavalcantei*. (**B**) Tertiary veins mixed percurrent, *Scleronema grandiflorum*.

We observed two types of leaf tertiary vein patterns, alternate percurrent (Figure 2A), and mixed percurrent (Figure 2B). In *Aguiaria* and *Scleronema*, the pattern is always mixed percurrent. In *Catostemma*, both patterns are present, and were consistent in each species analyzed. Because it is an easily identifiable characteristic, even in species with leaves of leathery consistency and barely evident veins, it helps to separate some groups of species of this genus. *Catostemma* species with the mixed percurrent pattern include *C. albuquerquei* Paula, *C. durifolium* W.S. Alverson, *C. fragrans*, and *C. milanezii* Paula; the remaining eight species in the genus are alternate percurrent. The leaves of seedlings of known species, although they may differ in the number of leaflets, indumentum, shape, and size, show the same pattern of tertiary veins as the leaves of adult individuals. Although the morphology of seedling leaves of eight species in the genus remains unknown, venation patterns likely will facilitate identification of samples collected in the future.

### 3.5. Flower and Inflorescence Morphology

**Figure 3:**
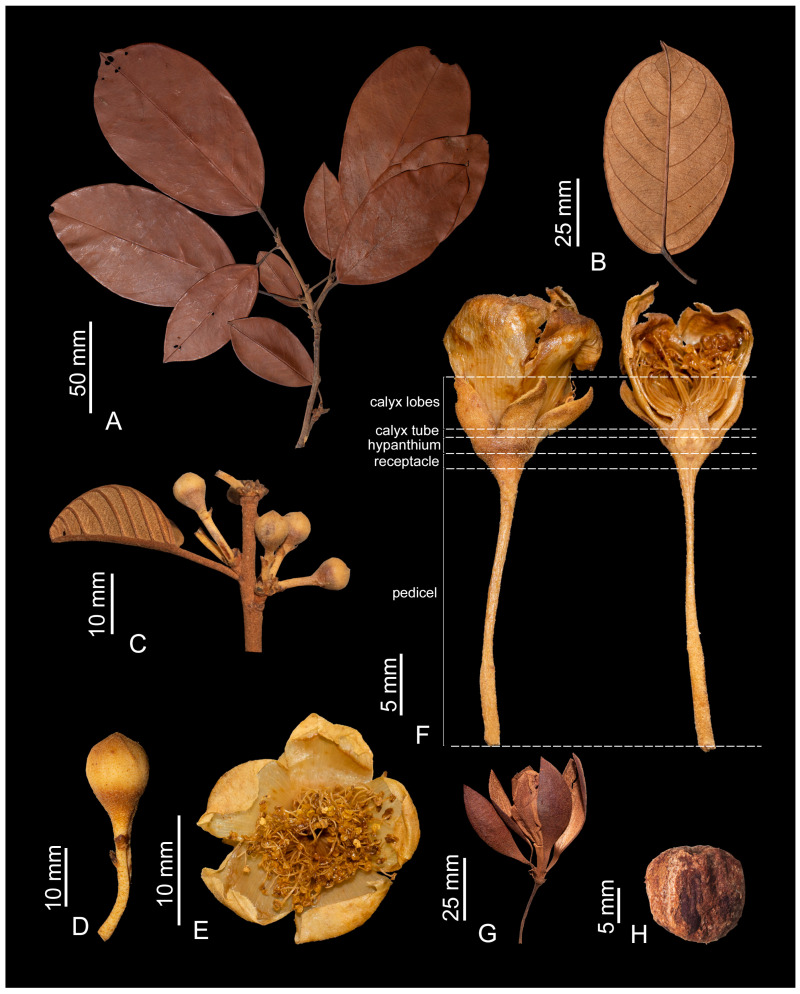
*Aguiaria excelsa*. (**A**) Sterile branch; (**B**) leaflet (abaxial surface); (**C**) flowering branch; (**D**) flower bud; (**E**) flower in front view; (**F**) flower in longitudinal section with details of the pedicel, receptacle, hypanthium, and calyx; (**G**) fruit; (**H**) seed. (Figures (**A**,**B**,**G**,**H**) based on *Ducke s.n.* and Figures (**C**–**F**) on *Ferreira et al. 939*. Photos by C.D.M. Ferreira.

**Figure 4:**
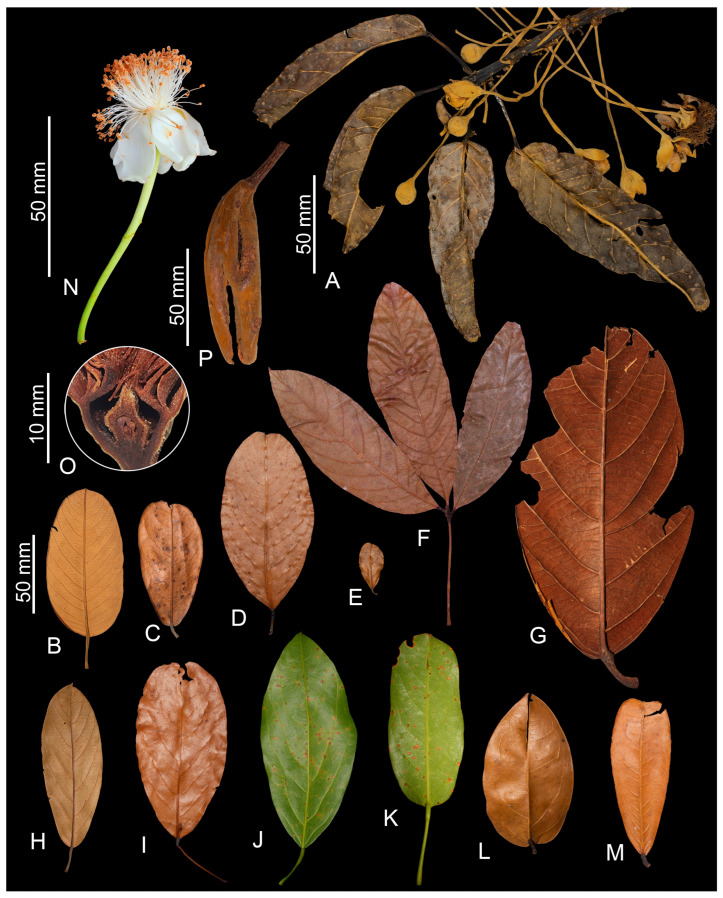
Species of *Catostemma*. (**A**) Flowering branch, *C. lanceolatum*; (**B**) leaflet (abaxial surface), *C. albuquerquei*; (**C**) leaflet (abaxial surface), *C. cavalcantei*; (**D**) leaflet (abaxial surface), *C. commune*; (**E**) leaflet (abaxial surface), *C. ebracteolatum*; (**F**) leaf (abaxial and adaxial surface), *C. digitatum*; (**G**) leaflet (abaxial surface), *C. durifolium*; (**H**) leaflet (abaxial surface), *C. fragrans*; (**I**) leaflet (abaxial surface), *C. grazielae*; (**J**) leaflet (abaxial surface), *C. milanezii*; (**K**,**L**) leaflet (abaxial surface), *C. sclerophyllum*; (**M**) leaflet (abaxial surface), *C. altsonii*; (**N**) flower, (**O**) longitudinal section of the hypanthium, *C. lanceolatum*; (**P**) fruit, *C. ebracteolatum*. Based on *Demarchi & Barcelos 872* (**A**,**N**,**O**), *Souza 45* (**B**), *Clarke et al. 12417* (**C**), *Gentry & Stein 47038* (**D**), *Fernandez & Yanez 447* (**E**), *Alverson et al. 397* (**F**), *Redden et al. 7286* (**G**), *Maguire et al. 54098* (**H**), *Mori et al. 17191* (**I**), *Ferreira et al. 859* (**J**), *Ferreira et al. 831* (**K**), *Cid Ferreira 9114* (**L**), *Mori et al. 8092* (**M**), and *Holst et al. 2759* (**P**). Dry materials (**A**–**I**,**L**,**M**,**O**), and fresh materials (**J**,**K**,**N**). Photos by C.D.M. Ferreira (**A**–**E**,**G**–**M**,**O**,**P**), W.S. Alverson (**F**), and L. Demarchi (**N**).

**Figure 9:**
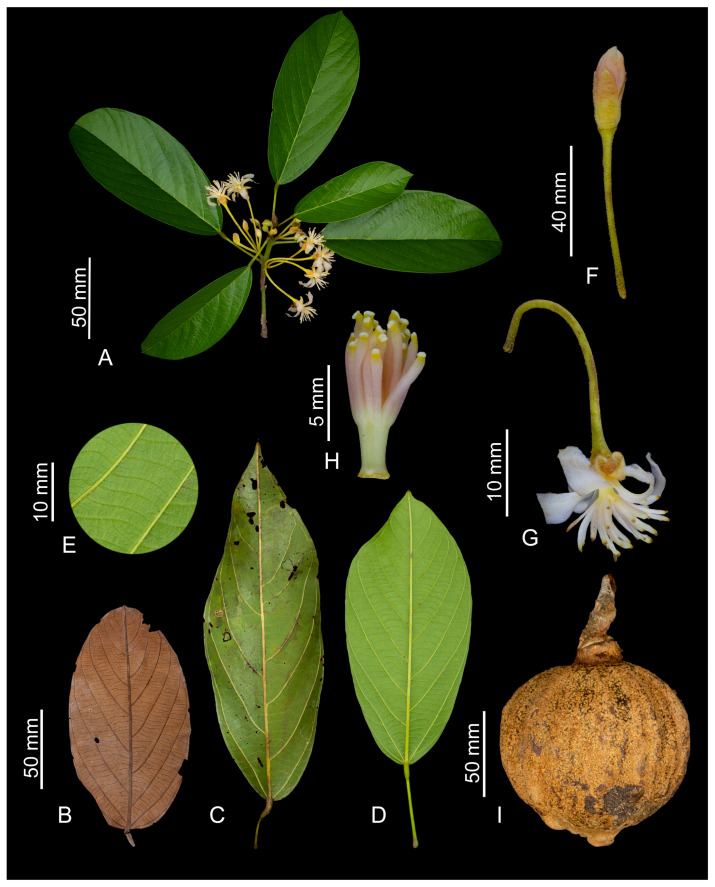
Species of *Scleronema*. (**A**) Flowering branch; (**B**) leaflet (abaxial surface), *S. praecox*; (**C**). leaflet (abaxial surface), *S. spruceanum;* (**D**) leaflet (abaxial surface), *S. grandiflorum*; (**E**) detail of tertiary veins; (**F**) flower bud; (**G**) flower; (**H**) androecium; (**I**) immature fruit, *S. grandiflorum*. Based on *Ferreira et al. s.n.* (**A**,**D**–**H**), *Drees 13* (**B**), *Ferreira et al. 837* (**C**), and *Ferreira & Viana 870* (**I**). Dry material (**B**), and fresh material (**A**,**C**–**I**). Photos by C.D.M. Ferreira.

The flowers of the Catostemma clade are grouped into fasciculate inflorescences in the leaf axils (Figure 3C, Figure 4A and Figure 9A). The number of flowers per inflorescence varies among species and also within a single species, thus it does not serve to differentiate taxa. Flowers are pedicellate, dichlamydeous, gamosepalous, and dialypetalous. The pedicel usually bears three bracteoles along its length (Figure 3D), except in the aptly named *Catostemma ebracteolatum* Steyerm. Bracteole size, shape, indumentum, and/or position on the pedicel (on the apical and/or median portion) can aid in identifying some species. However, depending on the phenological stage of the specimen, bracteoles are deciduous, in which case it is necessary to observe the scars that indicate the occurrence of these small structures. Calyces are variable: always 5-lobed in *Aguiaria*, 2–4-lobed in *Catostemma* and 3–5-lobed in *Scleronema*. Corollas have 5 petals, which are white in *Aguiaria* and *Catostemma*, and white to slightly pinkish or reddish in *Scleronema*. The androecium is monadelphous, with filaments fused at base into a column and free above. The indumentum of the column differs among genera: only *Aguiaria* has fasciculate trichomes, whereas in *Catostemma* and *Scleronema* the column is glabrous. Proportions of the column also differ: wider than long in *Aguiaria*, wider than long or rarely with similar length and width in *Catostemma*, and longer than wide in *Scleronema*. Number and relative girth of the free filaments also differs: 150–280 slender filaments in *Aguiaria*, 50–500 slender filaments in *Catostemma*, and 12–30 thickened filaments in *Scleronema*. Ovaries are superior, with 5 locules and 6(–7) ovules per locule in *Aguiaria*, and with (2–)3(–4) locules and usually 2 ovules per locule in *Catostemma* and *Scleronema*.

The flowering period of species in the clade is generally sporadic and asynchronous, including for individuals within a species. It rarely occurs in regular months but commonly occurs at intervals of 2–3 years, as observed by us and Paula [6].

An important characteristic observed in the Catostemma clade is its perigynous flowers (Figure 3F), not observed in other genera of the Adansonieae tribe (*Adansonia* and *Cavanillesia*) and the Bombacoideae tribe. We observed that the size and indumentum of the hypanthium and calyx tube are of great importance for the recognition of genera and species. The indumentum on the adaxial surface of the calyx tube aids in the differentiation of the three genera, namely fasciculate trichomes in *Aguiaria*, simple trichomes in *Scleronema*, and commonly glabrous in *Catostemma* (except for *C. lanceolatum*, which sometimes has sparse, simple trichomes).

### 3.6. Fruit and Seed Morphology

The Catostemma clade presents two types of fruits, capsules and woody berries. Both *Aguiaria* and *Catostemma* have capsules, but they differ significantly. Mature *Aguiaria* fruits are partially dehiscent in a unique way. The seed(s) remains attached to all the original parts of the fruit, including the columella, but its five thin valves detach except at their bases, creating a set of wings that aid dispersal (Figure 3G). In contrast, *Catostemma* has 2–4 thick valves (Figure 4P) that open partially along their sutures, such that the fruits fall with the seed(s) entirely or mostly enclosed by the valves. The fruit of *Scleronema* is an indehiscent woody berry (Figure 9I). All three genera feature an endocarp with a spongy texture, though this is only evident in *Aguiaria*, where it envelops and firmly adheres to the seed. In *Catostemma* and *Scleronema*, although present, the endocarp is scarce and difficult to visualize.

These genera typically produce one seed per fruit. Mature seeds of *Aguiaria* are smaller (about 1 × 1 cm) and those of *Catostemma* and *Scleronema* are larger (always > 2×2 cm). *Catostemma* is the only genus with arillate seeds. The aril has a pasty to oily-solid and consistency, yellowish, orange, or reddish in color, and is usually about 2 mm thick (Elio Sanoja, pers. comm.). After undergoing desiccation for preservation in an herbarium process, only a thin and fragile layer, without solid content, remains around the seed.

### 3.7. Taxonomic Treatment

Key to genera and species of the Catostemma clade

1. Trees with flying buttresses; branches, petioles, and pedicels with dentate-lepidote trichomes; staminal columns with fasciculate trichomes; ovaries 5-locular, with 6(–7) ovules per locule; fruits capsular, partially dehiscent, with 5 persistent valves that act as wings … ***Aguiaria excelsa***

1’. Trees without buttresses; branches, petioles, and pedicel with other types of trichomes; staminal column glabrous; ovary 2–4-locular, with 2(–3) ovules per locule; fruits capsular and partially dehiscent, with 2–4 deciduous valves that mostly surround the seed(s) and do not spread and become wing-like, or indehiscent woody berries … 2

2. Staminal columns with 12–30 relatively thick free filaments; ovaries globose to oblate, or ovoid; fruits indehiscent woody berries; seeds not arillate (***Scleronema***) … 3

2’. Staminal columns with 50–500 relatively slender free filaments; ovaries pyriform; fruits partially dehiscent capsules with (2–)3(–4) deciduous valves; seeds arillate (***Catostemma***) … 5

3. Branches, petioles, and stipules with fasciculate trichomes; stipule apices acuminate; abaxial surfaces of leaflets of adult trees without evident stomata; bracteole apices acuminate; petals white to slightly pinkish … ***Scleronema grandiflorum***

3’. Branches, petioles, and stipules with stellate-rotate trichomes; stipule apices acute or attenuate; abaxial surfaces of leaflets of adult trees with evident stomata; bracteole apices acute; petals reddish … 4

4. Branches and petioles glaucous; stipules ca. 0.2 × 0.1 cm, apices attenuate; leaflets of adult trees coriaceous, their abaxial surfaces glabrous or with fasciculate trichomes on the veins; bracteoles ca. 0.1 × 0.1 cm; ovaries ovoid … ***Scleronema spruceanum***

4’. Branches and petioles not glaucous; stipules ca. 0.8 × 0.3 cm, apices acute; leaflets of adult trees chartaceous, their abaxial surfaces with stellate-rotate trichomes; bracteoles ca. 0.7 × 0.2 cm; ovaries globose to oblate … ***Scleronema praecox***

5. Leaves with tertiary veins mixed percurrent … 6

5’. Leaves with tertiary veins alternate percurrent …9

6. Stipules ca. 0.8 cm wide; leaflets of adult trees bullate; pedicels 8.1–9.6 cm long; hypanthia 0.3–0.5 × 0.9–1 cm; calyx tubes 0.6–0.8 × 1.4–1.5 cm; staminal columns 0.7–0.8 cm wide; free filaments 450–500; ovaries 4-locular, with 3 ovules per … ***Catostemma durifolium***

6’. Stipules to ca. 0.4 cm wide; leaflets of adult trees not bullate; pedicels 0.7–3.8 cm long; hypanthia 0.05–0.3 × 0.3–0.5 cm; calyx tubes 0.05–0.4 × 0.4–0.7; staminal columns to ca. 0.3 cm wide; free filaments to 140; ovaries 3-locular, with 2 ovules per locule … 7

7. Leaflets of adult trees oblong, rarely slightly ovate, their apices rounded or slightly emarginate, margins revolute; calyces 3-lobate … ***Catostemma albuquerquei***

7’. Leaflets of adult trees obovate, oblanceolate or elliptic, their apices acuminate, emarginate or retuse, margins not revolute or only slightly revolute; calyces 2-lobate … 8

8. Leaflets of adult trees obovate to oblanceolate, rarely elliptic, apices retuse or emarginate, mucronate; pedicels 2.2–3.8 cm long; bracteole apices obtuse or acute; hypanthia 0.4–0.5 cm wide, their adaxial surface with fasciculate trichomes arranged in longitudinal rows; calyx tubes 0.6–0.7 cm wide, the lobes 0.8–0.9 cm long, apices rounded to obtuse; petals 1.6–1.8 × 0.4–0.7 cm; innermost filaments with fasciculate trichomes; styles 1.2–1.5 cm long … ***Catostemma fragrans***

8’. Leaflets of adult trees elliptic to obovate, apices acuminate; pedicels (0.7–)1.1–1.7 cm long; bracteole apices cuspidate; hypanthia ca. 0.3 cm wide, their adaxial surface glabrous; calyx tubes ca. 0.4 cm wide; calyx lobes 0.5–0.6 cm long, apices acuminate; petals ca. 1.1 × 0.3–0.4 cm; innermost filaments glabrous; styles 0.8–1 cm long … ***Catostemma milanezii***

9. Branches and petioles with fasciculate trichomes … 10

9’. Branches and petioles glabrous … 11

10. Stipule apices acuminate, their adaxial surfaces glabrous; leaflets of adult trees with revolute margins; petioles and calyces with fasciculate trichomes, flowers 3-bracteolate; calyces 4-lobate, the lobes 0.4–0.5 cm wide, apices acute; ovaries 3-locular, stigmas trifid … ***Catostemma cavalcantei***

10’. Stipule apices attenuate to caudate, their adaxial surfaces with glandular trichomes; leaflets of adult trees with margins not revolute; petioles and calyces with short and long fasciculate trichomes arranged in longitudinal rows; flowers ebracteolate; calyces 2-lobate, the lobes 0.9–1.1 cm wide, apices rounded; ovaries 2-locular, stigmas bifid … ***Catostemma ebracteolatum***

11. Leaves of adult trees (3–)5(–7)-foliolate; petioles 6–21 cm long; immature fruits ca. 6 cm wide, obovoid … ***Catostemma digitatum***

11’. Leaves of adult trees unifoliolate, sometimes trifoliolate; petioles (0.8)1.1–8.7(–9.6) cm long; immature fruits 2.4–4.9 cm wide, fusiform, obloid or slightly ovoid … 12

12. Pedicels ebracteolate; ovaries 2-locular, stigmas bifid … ***Catostemma ebracteolatum***

12’. Pedicels 3-bracteolate; ovaries 3-locular, stigmas trifid … 13

13. Leaves of adult trees trifoliolate … ***Catostemma commune***

13’. Leaves of adult trees unifoliolate … 14

14. Pedicels (1.3–)1.7–2.8 cm long, with fasciculate and dendritic trichomes; abaxial surfaces of bracteoles with fasciculate and dendritic trichomes … ***Catostemma commune***

14’. Pedicels 0.4–0.7(–1.1) or 3.4–8.6 cm long, with only fasciculate trichomes; abaxial surfaces of bracteoles only with fasciculate trichomes … 15

15. Pedicels 0.4–0.7(–1.1) cm long; bracteole apices acute; calyx lobes 0.4–0.7 × 0.3–0.5 cm; petals 0.7–0.9 × 0.3–0.4 cm … ***Catostemma grazielae***

15’. Pedicels 3.4–8.6 cm long; bracteole apices acuminate, cuneate, obtuse or rounded; calyx lobes 0.7–1.5 × 0.5–1.1 cm; petals 1.2–2.5 × 0.5–1.7 cm … 16

16. Bracteoles apices rounded; calyces 2-lobate; petals 0.9–1 cm wide … ***Catostemma altsonii***

16’. Bracteoles apices acuminate, cuneate or obtuse; calyces 3-lobate; petals 0.5–0.6 cm or 1.2–1.7 cm wide … 17

17. Leaflets of adult trees lanceolate to narrowly ovate; pedicels 7.1–8.4 cm long; bracteoles 0.3–0.8 × 0.2–0.4 cm, filiform, apices acuminate, the midvein of the adaxial surface with simple trichomes; hypanthia ca. 0.3 × 0.6 cm; calyx lobes ca. 1.5 × 0.6–1.1 cm, apices acute, the adaxial surface with simple trichomes; petals 2.1–2.5 × 1.2–1.7 cm; free filaments 420–450, ca. 1.8 cm long … ***Catostemma lanceolatum***

17’. Leaflets of adult trees oblong or obovate to lanceolate; pedicels 3.4–5 cm long; bracteoles ca. 0.1 × 0.1 cm, triangular, apices obtuse or cuneate, their abaxial surface with fasciculate trichomes; hypanthia 0.1–0.2 × 0.3 cm; calyx lobes 0.7–0.8 × 0.5–0.6 cm, apices obtuse, the adaxial surface glabrous; petals ca. 1.2 × 0.5–0.6 cm; free filaments 120–140, 0.6–0.7 cm long … ***Catostemma sclerophyllum***

***Aguiaria*** Ducke Ann. Acad. Brasil. Sci. 7(4): 329. 1935. *Aguiaria excelsa* Ducke

Trees unarmed, with flying buttresses. Trunk straight, bark brownish to reddish, striated with conspicuous striations, squamous in small irregular plates; crown broad; branches verticillate, plagiotropic. Stipules deciduous, triangular. Leaves of seedling and adult trees unifoliolate, petiolate, pulvinate, inarticulate; tertiary veins mixed percurrent. Inflorescences axillary, fasciculate; bracteoles 3, triangular, deciduous. Flowers pedicellate; calyces 5-lobed apically, gamosepalous; corollas dialypetalous, with 5 white petals; stamens numerous, their filaments fused at base into a cylindrical column that is wider than long, the free upper portions of the filaments slender; anthers monothecous, reniform; ovaries pyriform, 5-locular with 6(–7) ovules per locule, superior, perigynous, stigma 5-fidous. Fruits capsular, partially dehiscent, with 5 spreading valves that remain attached only at base, obovoid, 1(–2)-seeded, globose or obovoid, the seeds immersed in spongy endocarp tissue.

This is a monospecific genus, represented only by *A. excelsa*, which is restricted to the municipality of São Gabriel da Cachoeira in the upper Rio Negro region, Amazonas, Brazil. The fruit of *Aguiaria*, an incompletely dehiscent capsule with persistent valves, is unique among representatives of the clade. To our knowledge, there are no records of fruits with the same morphological adaptations for dispersal among other Eudicots. When mature, its valves assume a patent position, with the spongy endocarp firmly attached to the seed, persisting together with the columella. In this conformation, the fruit has a “propellor” or “wind vane” aspect, with similar utility. Its valves assume the function of wings, which carry the fruit to distances and directions guided by the wind. The specificity of this fruit was described by Ducke [5] and recently reaffirmed by Cardoso et al. [16].

***Aguiaria excelsa*** Ducke, Ann. Acad. Brasil. Sci. 7(4): 329. t.1. 1935. Type: BRAZIL. Prope São Gabriel. s/d, *C. Lakó s.n.* [*p.p.* only fruits] (lectotype, designated by Ferreira et al. [17]: K [photo]!; isolectotypes: INPA [fragment]!, RB [fragment]!, US [photo]!).

Trees 30–50 m tall. Branches not glaucous, densely covered with brown, dentate-lepidote trichomes. Stipules ca. 0.3 × 0.1 cm, apices attenuate, both surfaces not glaucous, the adaxial surface densely covered with brown, dentate-lepidote trichomes, the abaxial surface sparsely covered with brown, dentate-lepidote trichomes, the apices glabrous. Seedling leaves unifoliolate, petioles (5.9–)7.4–11.2 cm long, sparsely covered with brown, glandular trichomes, leaflets 22.3–27.4 × 8.5–11 cm, chartaceous, oblong, margins entire, bases rounded, apices caudate and mucronate, both surfaces sparsely or densely covered with brown, glandular trichomes, without evident stomata. Leaves of adult trees unifoliolate, petioles (1.9–)2.8–4.1 cm long, not glaucous, densely covered with brown, dentate-lepidote trichomes, leaflets (7.8–)9.3–14.2 × (5.1–)6.1–7.4 cm, chartaceous, elliptic to obovate, margins slightly revolute, bases rounded, apices acuminate and mucronate, the adaxial surface glabrous or sparsely covered with hyaline, dentate-lepidote and glandular trichomes, the abaxial surface densely with brown, dentate-lepidote and glandular trichomes, without evident stomata. Inflorescences 2–5-fasciculate; 3-bracteolate; bracteoles ca. 0.2 × 0.2 cm, triangular, apices acute to attenuate, the adaxial surface glabrous, the abaxial surface densely covered with brown, dentate-lepidote trichomes, the apical and/or median portions sometimes glabrous. Floral pedicels (2–)2.6–3.5 cm long, densely covered with brown, dentate-lepidote trichomes, hypanthia ca. 0.1 × 0.4 cm, the adaxial surface glabrous; calyx tubes ca. 0.2 × 0.7 cm, 5-lobate, lobes 0.7–0.9 × 0.4–0.5 cm, lobe apices acute, the adaxial surface of the tube and lobes densely covered with hyaline, fasciculate trichomes; abaxial surfaces of the hypanthium and calyx with the same indument of the pedicel; petals 1.2–1.6 × 0.6–1 cm, obovate, apices rounded, the basal portion of the adaxial surface densely covered with hyaline, simple, and fasciculate trichomes, the apical portion of the abaxial surface densely covered with hyaline, fasciculate trichomes; staminal columns 0.1–0.2 × ca. 0.3 cm, densely covered with hyaline, fasciculate trichomes, free filaments 150–280, each 0.8–1 cm long, glabrous; ovaries 0.3–0.4 × 0.3–0.4 cm, densely covered with hyaline to brown, fasciculate trichomes; style ca. 1.1 cm long, glabrous; stigmas glabrous. Fruits 3.1–4.5 × 1.2–2.1 cm, densely covered with brown, fasciculate trichomes; seed ca. 1.1 × 1.1 cm.

*Specimens Examined:* BRAZIL. Amazonas. São Gabriel da Cachoeira. 24 Oct 1932, st., *A. Ducke s.n* (RB); 16 Nov 1936, fl., *A. Ducke s.n* (IAN, INPA, NY, RB); 12 June 1936, fr., A. Ducke s.n. (F, NY, RB); 1 Nov 2021, st., *C.D.M. Ferreira et al. 846* (RB); 1 Nov 2021, st., *C.D.M. Ferreira et al. 847; 849* (RB); Itacoatiara Mirim, 31 Oct 2021, seed., *C.D.M. Ferreira et al. 839* (RB); 19 Nov 2022, fl., *C.D.M. Ferreira et al. 939* (RB); Camanaus, 1 Nov 2021, st., *C.D.M. Ferreira et al. 844*; *845* (RB).

*Common names: duraque, duraka* (possibly Arawak).

*Distribution and Habitat:* Restricted to the municipality of São Gabriel da Cachoeira in the region of the upper Rio Negro, Amazonas, Brazil, where it occurs on poorly drained, sandy soils, usually close to *igarapés* (an Amazonian waterway, like a stream or creek, typically a tributary to larger rivers), but in places not subject to seasonal flooding, at elevations of 60–80 m.s.m. (Figure 5).

*Phenology:* Flowering specimens were collected in November and fruiting specimens in June.

*Conservation Status:* Existing herbarium collections indicate that this species is known from fewer than five localities and has a restricted distribution, not exceeding 5000 km^2^ of Extent of Occurrence (EOO). Although there is historical use of its wood for building houses [5], our fieldwork in the São Gabriel da Cachoeira region observed numerous individuals at various stages of development, suggesting that the harvesting of this species likely occurred or occurs sporadically and in a planned manner. Our conversations with the local, indigenous residents made it clear that they hold this species in respect and value it for the construction of their *malocas* (large structures for living and ritual purposes) due to its significant durability and strength. They also informed us that the species occurs along the Rio Negro, from São João to the mouth of the Içana River, respectively, southeast and northwest of the city of São Gabriel da Cachoeira, a distance of approximately 160 km in a straight line. Unfortunately, logistic difficulties permeate this region, predominantly composed of indigenous territories, and access is only possible by boat. A significant infrastructure, beyond our means, was required to sustain an expedition in the region, so it was not possible to conduct collections along the entire length of the potential habitat. Neither could the total distribution of the species be confirmed with herbarium data. Therefore, since it is not possible to determine the actual area of occurrence nor the existence of direct or indirect threats to the known populations of this species, *A. excelsa* is herein considered DD (Data Deficient).

*Notes:* Vegetatively, *Aguiaria* can be distinguished from other genera of the Catostemma clade by its indument of dentate-lepidote trichomes. It also is the only genus with flying buttresses in adult individuals. Regarding the reproductive structures, the free filaments are slender and similar in number to most species of *Catostemma*, but its staminal column is covered by fasciculate trichomes (vs. the other species of the clade, which are glabrous).

***Catostemma*** Benth., London J. Bot. 2: 365. 1843. London J. Bot. 2: 365. 1843. *Catostemma fragrans* Benth.

Trees (10–)15–40(–50) m tall, unarmed, without buttresses. Trunks straight, bark brownish to reddish, striated with inconspicuous greenish striations, with visible lenticels; crown globose and narrow, branches verticillate, plagiotropic, usually glaucous and glabrous, sometimes with fasciculate trichomes. Stipules deciduous, triangular, with apices acuminate, attenuate or rarely acute, attenuate to caudate or cuspidate, the indumentum of fasciculate or glandular trichomes, rarely glabrous. Leaves of seedling and adult trees unifoliolate, or sometimes compound digitate 3–5(–7)-foliolate, petiolate, pulvinate, inarticulate; petiolules of seedling and adult trees absent or 0.2–2 cm long, articulate, glabrous; leaflets obovate, elliptic, oblong, oblanceolate, elliptic to obovate, lanceolate to narrowly ovate, obovate to oblanceolate, elliptic to oblong, elliptic to obovate, or lanceolate, the basal leaflets of digitately compound leaves asymmetric, usually coriaceous, chartaceous or chartaceous to coriaceous, rarely membranaceous, or membranaceous to chartaceous, rarely bullate (*C. durifolium*), the tertiary veins mixed percurrent or alternate percurrent. Inflorescences fasciculate, axillary; bracteoles 3, rarely absent, triangular, rarely filiform, deciduous. Flowers pedicellate; calyces 2–3(–4)-lobed apically, gamosepalous; corollas dialypetalous, petals 5, white; staminal filaments fused in at base into a cylindrical column that is usually wider than long or with similar length and width, rarely longer than wide (*C. commune*), glabrous, free filaments (50–)72–500, slender, glabrous (or rarely, in *C. fragrans*, the innermost filaments with fasciculate trichomes); anthers monothecous, reniform; ovaries pyriform, (2–)3(–4)-locular with 2(–3) ovules per locule, superior, perigynous, densely covered with brown, fasciculate trichomes, styles glabrous or rarely with fasciculate trichomes (*C. durifolium* and *C. fragrans*), stigmas (2–)3(–4)-fid, glabrous. Fruits capsules, partially dehiscent, with (2–)3(–4) valves, fusiform, obovoid, obloid, obovoid to ellipsoid, obovoid to fusiform, fusiform to ellipsoid, fusiform to slightly ovoid, or obloid to slightly obovoid, densely covered with brown, fasciculate trichomes, 1(–2)-seeded, the seeds ellipsoid, obloid, fusiform, fusiform to ellipsoid, or obovoid to ellipsoid, arillate, and immersed in scarce, spongy endocarp.

*Catostemma* is a widely distributed genus in Amazonian rainforest of Brazil, Colombia, French Guiana, Guyana, Suriname, and Venezuela, with a single species (*C. digitatum*) in the inter-Andean watersheds of Colombia. The species are found on sandy, clay, or sandy-clay soils, usually in well-drained areas subject to seasonal flooding, typically on flat terrain (rarely with a steep slope: *C. digitatum* and *C. durifolium*) at elevations of 10–700 m.s.m., and rarely at elevations above 1000 m.s.m. (*C. commune*, *C. digitatum*, and *C. durifolium*). It has a vegetative morphology like *Scleronema*. Both genera have unifoliolate leaves in adult individuals, except for *C. digitatum*, which always has digitate leaves, and *C. commune*, which may have trifoliate leaves on some branches. Both patterns of tertiary veins, described above, are observed in *Catostemma*, but *Scleronema* only appears to have the mixed-percurrent pattern. Consequently, an examination of reproductive characters is necessary to differentiate the two genera. Species of *Catostemma* have a staminal column that is wider than it is long or with similar length and width, rarely longer than wide (vs. staminal columns longer than wide); 50–500 slender filaments (vs. 12–30 thickened filaments); pyriform ovaries (vs. globose to oblate, or ovoid), and capsular fruits (vs. woody berries).

***Catostemma albuquerquei*** Paula, Ci. & Cult. 21(4): 702. 1969. Type: BRAZIL. Amazonas. Manaus, estrada Manaus-Itacoatiara, Km 104, lado esquerdo, à 1700 m da margem da estrada, mata primária de terra firme, 9 May 1967, *Byron & Elias 67/44*, (holotype: INPA [4 sheets] [photo]!); isotype: BR [photo]!).

Trees ca. 25 m tall. Branches not glaucous, densely covered with brown, fasciculate trichomes. Stipules ca. 0.6 × 0.4 cm, apices acuminate, both surfaces not glaucous, the adaxial surface glabrous, the abaxial surface densely covered with brown, fasciculate trichomes. Seedling leaves not seen. Leaves of adult trees unifoliolate, petioles (1.6–)1.9–2.7 cm long, not glaucous, densely covered with brown, fasciculate trichomes, leaflets 8.6–11.3 × 4.2–5.6 cm, coriaceous, not bullate, oblong, rarely slightly ovate, margins revolute, bases rounded, apices rounded, or slightly emarginate, mucronate, the adaxial surface glabrous, the abaxial surface densely covered with brown, fasciculate trichomes and without evident stomata, tertiary veins mixed percurrent. Inflorescences 3–5-fasciculate, axillary, 3-bracteolate; bracteoles ca. 0.1 × 0.1 cm, triangular, apices acute, the adaxial surface glabrous, the abaxial surface densely covered with brown, fasciculate trichomes and without evident stomata. Floral pedicels 1.5–2 cm long, densely covered with brown, fasciculate trichomes; hypanthia ca. 0.1 × 0.3 cm; calyx tubes 0.05–0.1 × 0.4–0.5 cm, apices 3-lobate, lobes ca. 0.9 × 0.4 cm, lobe apices acute, the hypanthium and adaxial surfaces of the calyx glabrous, the abaxial surface with the same indument of the pedicel; petals ca. 1.3 × 0.5 cm, oblong to slightly obovate, apices obtuse, both surfaces glabrous; staminal columns ca. 0.3 × 0.3 cm, free filaments 50–72, 0.6–0.9 cm long, glabrous; ovaries ca. 0.3 × 0.2 cm, 3-locular, 2 ovules per locule; styles ca. 0.9 cm long, glabrous; stigmas trifid. Fruits 10.5–12 × 3–6 cm, fusiform, 1-seeded; seed 5.5–8 × 2.5–3.6 cm, ellipsoid to oblong.

*Specimens Examined:* Brazil. Amazonas. Manaus. Reserva Florestal Adolfo Ducke, 17 July 1968, fl., *J.A. Souza 45* (INPA, RB); estrada Manaus-Itacoatiara, km 106, 19 Sep 1965, fr., *W. Rodrigues & A. Loureiro 7164* (MG, INPA); Distrito agropecuário da SUFRAMA, 25 Sep 1986, st., *N.I. Ahmad et al. s.n.* (US); 21 Nov1989, fr., *A.P. da Silva s.n.* (US); 7 Jan 2003, st., *J.L.C. Camargo & A.E. Santos GPP30* (INPA).

*Common names: mamao-rana* (possibly Tupi).

*Distribution and Habitat: Catostemma albuquerquei* is endemic to the municipality of Manaus, Amazonas, Brazil, on well-drained clay soils not subject to flooding, at elevations of 70–100 m.s.m. (Figure 5).

*Phenology:* Flowering specimens were collected in July and fruiting specimens from September to May.

*Conservation Status:* A species with a restricted distribution, not exceeding 10 km^2^ of Area of Occupancy (AOO) and known from only two localities, only one of which is protected (Reserva Florestal Adolfo Ducke), while the other was not found in our field efforts (suggesting it may have been extirpated, especially due to its proximity to a road and constructions). Last collected in 1989, *C. albuquerquei* is herein considered CR (B1 ab [i, ii, iii]).

*Notes:* Morphologically like *C. cavalcantei*, due to its branches covered by fasciculate trichomes, and coriaceous leaflets with revolute margins. However, it differs by the abaxial surface of its leaflets that are densely covered by brown fasciculate trichomes (vs. glandular trichomes), mixed percurrent tertiary vein pattern (vs. alternate percurrent), 3-lobate calyces (vs. 4-lobate), and 50–72 free filaments (vs. 106–120).

***Catostemma altsonii*** Sandwith, Bull. Misc. Inform. Kew 1928(9): 366. 1928. Type: GUYANA. Macreba Falls. Kurupung River, Sep 1925, *Altson 391* (lectotype, designated by Ferreira et al. [17]: K [photo]!; isolectotype: K [photo]!).

Figure 4M

Trees 20–30 m tall. Branches usually glaucous, glabrous. Stipules ca. 0.6 × 0.4 cm, apices acuminate, both surfaces usually glaucous and glabrous. Seedling leaves not seen. Leaves of adult trees unifoliolate, petioles (0.8)–1.1–4.3 cm long, usually glaucous, glabrous, leaflets 11.6–17.3(–22.9) × 5.1–8.5 cm, coriaceous, not bullate, obovate or elliptic to oblong, margins entire or sometimes slightly revolute, bases slightly attenuate or rounded to slightly truncate, apices retuse and mucronate, the adaxial surface glabrous, midvein of abaxial surface sparsely covered with brown, glandular trichomes, without evident stomata, tertiary veins alternate percurrent. Inflorescences 3–7-fasciculate, axillary, 3-bracteolate; bracteoles ca. 0.2 × 0.1 cm, triangular, apices rounded, the adaxial surface at apex sparsely covered brown, fasciculate trichomes, the abaxial surface densely covered with brown, fasciculate trichomes and glabrous at apex. Floral pedicels (4.6–)6.1–8.6 cm long, densely covered with brown, fasciculate trichomes; hypanthia 0.05–0.1 × 0.4–0.5 cm; calyx tubes 0.2–0.3 × 0.5–0.7 cm, apices 2-lobate, lobes 1–1.2 × 0.7–0.8 cm, lobe apices rounded or acute, the hypanthium and adaxial surface of the calyx tube glabrous, the calyx lobes apex sparsely covered with hyaline, fasciculate and simple trichomes; the abaxial surfaces of the hypanthia, calyx tubes, and calyx lobes densely covered with the same indument of the pedicel; petals 1.8–2.2 × 0.9–1 cm, obovate to oblanceolate, apices rounded and both surfaces with hyaline, fasciculate trichomes; staminal columns ca. 0.3 × 0.3–0.4 cm, free filaments 200–280, 1–1.3 cm long, glabrous; ovaries ca. 0.2 × 0.2–0.3 cm, 3-locular with 2 ovules per locule; styles 1.2–1.5 cm long, glabrous; stigmas trifid. Immature fruits ca. 13.1 × 3.2 cm, fusiform, 1-seeded; immature seed ca. 3.5 × 1.4 cm, fusiform.

*Specimens Examined:* GUYANA. Cuyuni-Mazaruni Region. 2 July 2004, fl. and fr., *H.D. Clarke et al. 12417* (MO, NY); Mazaruni-Potaro. 15 Aug 1976, fl., *S. Mori et al. 8092* (AMAZ, NY); Mt. Ayanganna. 3 Aug 1960, fl., *S.S. Tillett et al. 45025* (MO, NY); upper Mazaruni river basin, 17 Aug 1960, fr., *S.S. Tillett et al. 45884* (MO, NY); Membaru Creek. 16 Feb 1939, fr., *A.S. Pinkus 238* (MO); Selvas del Río Caroní. 21 Sep 1946, fl., *F. Cardona 1648* (NY); VENEZUELA. Amazonas. Autana. 30 Jan 1997, fl., *A. Castillo 4567* (MO); Rio Negro. Rio Pasimoni, Apr 1991, st., *J. Velazco 2020* (MO); Bolívar. Cedeño. 6 Mar 1992, fl., *B.M. Boom & E. Marin 10323* (MO).

*Common names: baromalli* (possibly Arawak).

*Distribution and Habitat:* Occurs in Cuyuni-Mazaruni, Mazaruni-Potaro, Ayanganna Mountain, and Membaru Creek in Guyana; and Amazonas, Rio Negro, and Bolivar states in Venezuela. Grows on well-drained clay soils, not subject to flooding, at elevations of 100–900 m.s.m. (Figure 5).

*Phenology:* Flowering specimens were collected from March to July and fruiting specimens from February to July.

*Conservation Status:* A widely distributed species, EOO greater than 20,000 km^2^, and several known localities. Despite its wide geographic distribution, *C. altsonii* lacks recent collections (the latest being from 1997) and has no documented occurrences within protected areas. Given these data deficiencies, *C. altsonii* is herein considered DD.

*Notes:* Morphologically like *C. cavalcantei*, with an abaxial leaflet surface densely covered by glandular trichomes, tertiary veins that are alternate percurrent, and bracteoles with rounded apices, but distinguished by the glabrous abaxial surface and length (0.6 cm) of the stipules (vs. densely covered by fasciculate trichomes and ca. 0.4 cm long), 2-lobate calyces (vs. 4-lobate), and 200–280 free filaments (vs. 106–120).

***Catostemma cavalcantei*** Paula, Ci. & Cult. 21(4): 701. 1969. Type: BRAZIL. Amazonas. Manaus, rio Preto, mata de caatinga, solo arenoso, 11 Oct 1947, *R. Fróes 22802* (holotype: IAN [photo]!).

*Catostemma clarkii* Steyerm., Ann. Missouri Bot. Gard. 74: 638. 1987. Type: VENEZUELA. Amazonas. Mari’s bana (low Amazon caatinga), 10.8 km NE of San Carlos on road to Solano, 19 Aug 1981, *H.L. Clark 8126* (holotype: MO [photo]!; isotypes: INPA!; NY [photo]!; VEN [photo]!) **Syn. nov.**

*Catostemma pubistylum* Steyerm., Ann. Missouri Bot. Gard. 74: 643. 1987. Type: VENEZUELA. Amazonas. IVIC main study site, 4.3 km NE of San Carlos de Río Negro, 119 m., 3 Aug 1978, *H.L. Clark & P. Maquirino 6742* (holotype: NY [photo]!; isotype: K [photo]!) **Syn. nov.**

*Catostemma sancarlosianum* Steyerm., Ann. Missouri Bot. Gard. 74: 644. 1987. Type: VENEZUELA. Amazonas. Mari’s bana (low Amazon caatinga), 10.8 km NE of San Carlos on road to Solano, 119m. 16 Aug 1981, *H.L. Clark & K. Clark 8117* (holotype: MO [photo]!; isotypes: INPA!; NY [photo]!; US [photo]!; VEN [2 sheets] [photos]!) **Syn. nov.**

Figure 4C

Trees 30–50 m tall. Branches usually glaucous, densely or sparsely covered with brown, fasciculate trichomes. Stipules ca. 0.4 × 0.3 cm, apices acuminate, both surfaces not glaucous, the adaxial surface glabrous, the abaxial surface densely covered with brown, fasciculate trichomes. Seedling leaves not seen. Leaves of adult tree unifoliolate, petioles (0.5–)0.9–2.3 cm long, usually glaucous, densely or sparsely with brown, fasciculate trichomes, sometimes glabrous, leaflets (4.8–)6.1–11.3(–15) × (2.1–)3.5–5.7(–6.1) cm, coriaceous, not bullate, obovate to oblanceolate, margins revolute, bases cuneate, rarely subcordate, apices emarginate and mucronate, the adaxial surface sparsely with brown, glandular trichomes, the abaxial surface densely covered with brown, glandular trichomes and without evident stomata, tertiary veins alternate percurrent. Inflorescences 3–7-fasciculate, axillary; 3-bracteolate; bracteoles ca. 0.1 × 0.1 cm, triangular, apices rounded, adaxially glabrous, the abaxial surface densely covered with brown, fasciculate trichomes. Floral pedicels (1.5–)2.5–3.7 cm long, densely covered with brown, fasciculate trichomes; hypanthia ca. 0.2 × 0.4 cm; calyx tubes ca. 0.1 × 0.5 cm, apices 4-lobate, lobes 0.7–0.8 × 0.4–0.5 cm, lobe apices acute, the hypanthium and the adaxial surface of the calyx tube glabrous, calyx lobes glabrous or sometimes sparsely with hyaline, fasciculate trichomes at apices; abaxial surfaces of the hypanthium, calyx tube, and calyx lobes with same indument as the pedicel; petals (0.9–)1.2–1.5 × (0.4–)0.6–0.8 cm, oblong or obovate, apices rounded and both surfaces with hyaline, fasciculate trichomes; staminal columns ca. 0.1 × 0.2 cm, free filaments 106–120, (0.7–)0.9–1.2 cm long, glabrous; ovaries ca. 0.3 × 0.2 cm, 3-locular, 2(–3) ovules per locule; style 0.9–1.2 cm long, glabrous; stigma trifid. Immature fruits 6.1–6.8 × 2.4–2.6 cm, fusiform, 1-seeded; immature seeds ca. 2.5 × 1.6 cm, obovoid to ellipsoid.

*Specimens Examined:* BRASIL. Amazonas. Manaus. Rio Preto, 8 Nov 1947, fl., *R.L. Fróes 22789* (MG, IAN); Barcelos. margem do Rio Aracá, 29 Oct 1952, fl., *R.L. Fróes & G. Addison 29139* (IAN); 29 Feb 1984, fr., *J.M.S. Miralha 58* (INPA, MO, US); Serra do Aracá. 27 Feb 1977, fr., *N.A. Rosa & M.R. Cordeiro 1690* (MG, MO); 3 Mar 1984, st., *W.A. Rodrigues et al. 10906* (INPA, RB); 28 Feb 1984, fr., *J. Pipoly et al. 6726* (MO); Santa Isabel do Rio Negro. Tapuruquara, 28 Oct 1971, fr., *G.T. Prance et al. 15646* (INPA, MO, US); São Gabriel da Cachoeira. 17 Sep 2010, st., *M.R.M. Amaral et al. 222* (EAFM); Roraima. Caracaraí. Rio Xeruini, 16 Apr 1974, st., *Pires 14001* (MG); COLOMBIA. Guainía, San Felipe. 14 Oct 2009, fr., *D. Cárdenas et al. 24049* (COAH); VENEZUELA. Amazonas. San Carlos de Río Negro. 19 Sep 1975, fr., *P.E. Berry 1471* (MO, VEN); 3 Apr 2000, st., *P.E. Berry & G. Aymard 7544* (MO); 3 Apr 2000, st., *P.E. Berry & G. Aymard 7551* (MO); 20 Sep 1983, st., *V. Kapos & E. Tanner 106* (MO); 22 Sep 1983, st., *V. Kapos & E. Tanner 139* (MO); 13 Aug 1981, fl., *H.L. Clark & P. Maquirino 8115* (COL, MO, QCNE); 19 Aug 1981, fl., *H.L. Clark 8128* (MO, NY); 19 Aug 1981, fl., *H.L. Clark 8132* (MO, NY); 14 Aug 1981, fl., *H.L. Clark & K. Clark 8109* (MO, VEN); 1 Jan 1979, fr., *H.L. Clark 6919* (MO, NY); 25 Jan 1985, fr., *B.M. Boom et al. 5349* (NY); 26 Mar 2004, st., *L. Marcano-Berti et al. 2004-014* (VEN).

*Common names: cardeiro-duro* (Portuguese); *tetón, tetón-banero* (likely Spanish).

*Distribution and Habitat:* Occurs in Amazonas, and Roraima states in Brazil, the department of Guainía in Colombia, and Amazonas state in Venezuela on well-drained sandy soils not subject to flooding, at elevations of 60–200 m.s.m. (Figure 5).

*Phenology:* Flowering specimens were collected from August to November and fruiting specimens from September to February.

*Conservation Status:* Because it is a widely distributed species with an EOO greater than 20,000 km^2^ and several known occurrence localities, *C. cavalcantei* is herein considered LC.

*Notes:* Morphologically like *C. altsonii* and *C. albuquerquei*; we discuss the main similarities and differences below.

With regard to the three names here proposed as new synonyms for *C. cavalcantei*, *C. clarkii* and *C. pubistylum* were treated as synonyms of *C. sancarlosianum* by Alverson & Steyermark [18] and Ferreira et al. [17]. After morphological analysis of the type specimens of these three species, we conclude that there are no significant differences with *C. cavalcantei*. Paula [6] described this species from individuals collected in the municipality of Manaus, Amazonas, Brazil. It is odd but possible that Steyermark, while preparing his treatment for the Flora of Venezuela, did not have access to specimens seen by Paula and did not see Paula’s publication, since he does not refer to it in his work.

***Catostemma commune*** Sandwith, Bull. Misc. Inform. Kew 1931(1): 51. 1931. Type: GUYANA. Moraballi Creek. Essequibo River, 25 Aug 1929, *Sandwith 116* (lectotype, designated by Ferreira et al. [17]: K [2sheets] [photos]!; isolectotype: NY [photo]!). VENEZUELA. Delta Amacuro. bosque pluvial, Este de Río Grande. Este-Noreste de El Palmar, cerca de los limites del Estado Bolívar, Nov 1995, *C. Blanco 495* (epitype, designated by Ferreira et al. [17]: NY!).

*Catostemma marahuacense* Steyerm., Ann. Missouri Bot. Gard. 74: 643. 1987. Type: VENEZUELA. Amazonas. Atabapo, Cerro Marahuaca. “Sima Camp,” south-central portion of forested slopes along eastern branch of Caño Negro, 3°43’N 65°31’W, 1140 m. 28 Feb–1 Mar 1985, *J.A. Steyermark & B. Holst 130878* (holotype: MO [photo]!; isotypes: NY [2 sheets][photos]!; US [photo]!; VEN [photo]!) **Syn. nov.**

Figure 4D

Trees 30–50 m tall. Branches usually glaucous, glabrous. Stipules 0.4–0.5 × 0.3–0.4 cm, apices attenuate, both surfaces not glaucous, the adaxial surface glabrous, the abaxial surface glabrous, rarely sparsely covered with hyaline fasciculate trichomes. Seedling leaves unifoliolate or trifoliolate; unifoliolate leaves with petioles (2.6–)4.1–6.1 cm long, glabrous, leaflets 21.9–25 × 6.5–7.9 cm, elliptic to slightly obovate, margins entire, not revolute, bases attenuate, apices acuminate, mucronate; trifoliolate leaves with petioles (16.9–)23.7–30.3 cm long, glabrous, without petiolules, leaflets 22.4–24 × 4.7–6.9 cm, elliptic to slightly obovate, margins entire, not revolute, bases attenuate, apices acuminate and mucronate, basal leaflets asymmetric; unifoliolate and trifoliolate leaves membranaceous to chartaceous, not bullate, both surfaces densely covered with brown, glandular trichomes, without evident stomata, the tertiary veins alternate percurrent. Leaves of adult trees unifoliolate or trifoliolate; unifoliolate leaves with petioles (1.1–)1.7–4.3(–8) cm long, usually glaucous, glabrous, leaflets 9.2–13.6 × 5.1–7.6 cm, obovate to oblanceolate or elliptic, bases cuneate or slightly truncate; trifoliolate leaves with petioles 5.8–8.4 cm long, usually glaucous, glabrous, without petiolules, leaflets 8.2–10.8 × 2.8–3.7 cm, obovate to oblanceolate, bases attenuate, basal leaflets asymmetric; both unifoliolate and trifoliolate leaves coriaceous, not bullate, margins entire, not revolute, apices retuse to emarginate and mucronate, both surfaces glabrous or densely covered with brown, glandular trichomes, without evident stomata, the tertiary veins alternate percurrent. Inflorescences 4–10-fasciculate, axillary, 3-bracteolate; bracteoles 0.2–0.3 × 0.2–0.3 cm, triangular, apices obtuse or rounded, the adaxial surface glabrous, the abaxial surface densely covered with brown, fasciculate and dendritic trichomes. Floral pedicels (1.3–)1.7–2.8 cm long, densely covered with brown, fasciculate and dendritic trichomes; hypanthia 0.2–0.3 × 0.3–0.4 cm; calyx tubes ca. 0.1 × 0.4 cm, apices 2–3-lobate, lobes 0.5–0.7 × 0.4–0.7 cm, lobe apices acute, the adaxial surface of the hypanthium and calyx tube glabrous, with calyx lobes apices sparsely with hyaline, fasciculate trichomes; abaxial surfaces of the hypanthium, calyx tube, and calyx lobes with same indument as the pedicel; petals 1.2–1.4 × 0.4–0.5 cm, obovate to oblanceolate, apices obtuse, both surfaces glabrous; staminal columns ca. 0.3 × 0.2 cm, free filaments 130–180, 0.7–0.8 cm long, glabrous; ovaries ca. 0.3 × 0.2 cm, 3-locular with 2 ovules per locule; styles 1.1–1.3 cm long, glabrous; stigmas trifid. Immature fruits 6.9–10 × 2.7–4.9 cm, fusiform or obloid, 1(–2)-seeded; immature seeds ca. 3.7 × 2–2.3. cm, obloid.

*Representative Specimens Examined:* BRAZIL. Amazonas. São Gabriel da Cachoeira. 15 Sep 2010, st., *M.R.M. Amaral et al. 188* (EAFM); COLOMBIA. Amazonas. 23 May 1997, st., *M. Sánchez et al. 2935* (COAH); Caquetá. s.d., st., *S. Bergeron & L. Román 684-463* (COAH, COL); Guainia. Puerto Inírida, 27 Mar 1996, seed., *A. Etter & E. Fernandes 97* (COAH); Vaupés. 4 Sep 2013, st., *D. Cárdenas et al. 44192* (COAH); 12 Feb 2013, st., *J.S. Barreto et al. 3248* (COAH); 25 June 2009, st., *S. Castro et al. 1748* (COAH); FRENCH GUIANA. 11 Mar 2009, seed., *D. Sabatier & J.-F. Molino 5539* (NY); Grand Inini River. 23 July 1990, st., *D. Sabatier & M.F. Prevost 3415* (MO); GUYANA. Port Kaituma. 25 Oct 1990, fr., *M. Polak et al. 60* (NY); Potaro-Siparuni. Kaieteur National Park, 11–19 Mar 1997, seed., *C.L. Kelloff & G. McKee 1207b* (MO); VENEZUELA. Amazonas. 2 June 1996, seed., *G. Aymard & E. Melgueiro 11222* (MO); Atabapo. 25 Feb 1985, fr., *J.A. Steyermark & B. Holst 130709* (MO, NY, VEN); 5 Nov 1988, seed., *R. Liesner 25819* (MO); Atures. May 1989, fr., *E. Foldats & J. Velazco 9194* (MO); Oct 1989, fr., *J. Velazco 574* (MO); Río Negro. 4 Dec 1984, fr., *B.M. Boom & A.L. Weitzman 5277* (MG, NY); Apr 1991, st., *J. Velazco 2065* (MO); 3 May 1984, fr., *A. Gentry & B. Stein 47115* (MO); San Carlos de Río Negro. 27 Mar 2000, seed., *P.E. Berry G. Aymard 7235* (MO); 23 Mar 2000, seed., *P.E. Berry & G. Aymard 7058* (MO); Bolivar. 11 Mar 1966, st., *F.J. Breteler 972* (MG); 2 Feb 1994, seed., *L. Salas TT-116* (MO); 22 Oct 1985, st., *B.K. Holst & R. Liesner 2422* (MO, VEN); 6 Apr 1967, fl., *J. Bruijn 1631* (MO); 5 Apr 1967, st., *J. Bruijn 1623* (MO); Caño Pablo. 9 May 1982, fr., *G. Morilo & R. Liesner 9039* (MO); Cedeño. 21 July 1992, st., *C. Knab & Denis 117* (MO); Gran Sabana. 11 Aug 2004, st., *E. Sanoja et al. 6870* (VEN); Feb 1986, seed., *A. Fernandez 1799* (MO); Heres. 12 May 1987, st., *G. Aymard 5772* (MO); Raul Leoni. 30 Oct –2 Nov 1988, seed., *G. Aymard & A. Fernandez 7189* (MO); Roseío. El Palmar, Jun–Jul 1961, st., *J.C. Sobrino 36* (MO); Sifontes. 11 Aug 1985, fr., *G. Aymard et al. 4153* (MO). See additional specimens examined in Appendix A.

*Common names: baraman*, *palo-de-cancho* (Spanish); common-baromalli (English); *guachiguamike*, *kemayú*, *quema*, *perepodek* (unknown); *jadaikania*, *jagarai* (Witoto); *hubuo*, *júbuo*, *unitajadio* (Mui); *zamauma*, *dová* (Puinave).

*Distribution and Habitat:* Occurs widely in Colombia, French Guiana, and Venezuela, but in Brazil it only occurs in Amazonas in the municipality of São Gabriel da Cachoeira. Grows on sand and clay soils, generally poorly drained and subject to seasonal flooding, or rarely on well-drained, non-flooded soils at high elevations. The elevational range is 40–1300 m.s.m. (Figure 6).

*Phenology:* Flowering specimens were collected from December to July and fruiting specimens from February to December.

*Conservation Status:* This is a widely distributed species with an EOO greater than 20,000 km^2^, several known occurrence localities and with some of these in protected areas, so *C. commune* is herein considered LC.

*Notes:* This species resembles *C. ebracteolatum* and *C. grazielae*, which also have coriaceous unifoliolate leaves with leaflets usually obovate to oblanceolate, densely covered by glandular trichomes on both sides. Leaflet length differs in *C. ebracteolatum*, 3.4–6.5 cm long (vs. 9.2–13.6 cm long in *C. commune*); in addition, the pedicels and calyces are densely covered by short and long, fasciculate trichomes that are arranged in longitudinal rows (vs. densely covered by fasciculate and dendritic trichomes of the same size *in C. commune*), and bracteoles are absent (vs. 3-bracteolate in *C. commune*). *C. grazielae* differs in its pedicel length, 0.4–0.7(–1.1) cm long (vs. (1.3–)1.7–2.8 cm long in *C. commune*) and it is densely covered by fasciculate trichomes of the same size; has smaller petals, 0.7–0.9 × 0.3–0.4 cm (vs. 1.2–1.4 × 0.4–0.5 cm in *C. commune*), fruits fusiform to slightly ovoid (vs. fusiform or obloid in *C. commune*). Sometimes, the leaves of adult individuals of *C. commune* are trifoliate and resemble those of *C. digitatum*. However, the compound leaves of *C. commune* are usually trifoliate (vs. (3–)5(–7)-foliolate in *C. digitatum*) and are usually accompanied by unifoliate leaves on the same branch (vs. usually only digitate leaves).

*Catostemma marahuacense* is here proposed as a new synonym for *C. commune*. Based on our analysis of the type specimens, we conclude that there are no significant morphological characteristics to separate it from *C. commune*.

***Catostemma digitatum*** J.D. Sheph. & W.S. Alverson, Brittonia 33(4): 587. 1981. Type: COLOMBIA. Antioquia. confluence of Quebrada La Tirana and Río Anorí, 3 km upriver (SW) from Planta Providencia, 9 Apr 1977, *W.S. Alverson et al. 397* (holotype: COL!; isotypes: HUA!; MO [2 sheets]!; MO [2 sheets]!; NY [2 sheets]!; WIS [2 sheets]!).

Figure 4F

Trees 25–30 m tall. Branches usually glaucous, glabrous. Stipules ca. 0.4 × 0.1 cm, apices attenuate, both surfaces not glaucous, the adaxial surface glabrous, the midvein and basal portion of abaxial surface densely covered with bifurcate hyaline trichomes. Seedling leaves 5-foliolate, petioles ca. 9.4 cm long, glabrous, without petiolules, leaflets 10.9–19.5 × 2.2–3.9 cm, membranaceous, not bullate, lanceolate, margins entire, not revolute, bases attenuate, apices caudate and mucronate, both surfaces densely covered with brown, glandular trichomes, without evident stomata, the tertiary veins alternate percurrent. Leaves of adult trees compound digitate, (3–)5(–7)-foliolate, petioles 6–21 cm long, not glaucous, glabrous, petiolules 0.2–2 cm long, glabrous, leaflets 9–40 × 2–10 cm, chartaceous to coriaceous, not bullate, elliptic to obovate, margins entire, not revolute, bases cuneate or attenuate, apices attenuate and mucronate, basal leaflets asymmetric, both surfaces densely covered with brown, glandular trichomes, the tertiary veins alternate percurrent. Flowers not seen. Immature fruits ca. 12 × 6 cm, obovoid, 1-seeded; immature seeds 5–7 × 2–4 cm, ellipsoid.

*Specimens Examined:* COLOMBIA. Antioquia. Anorí. Providencia, 10 Dec 1972, st., *D.D. Soejarto & E. Renteria 3585* (HUA); 22 June 1972, seed., *J. Denslow 1404a* (MO, NY); 22 June 1972, seed. and fr., *J. Denslow 1402* (COL, MO); Boyacá. Reserva Natural de Aves El Paujil. 12 Jan 2007, fr., *A. Aldana et al. 403* (COL); 16 Jan 2007, st., *A. Aldana et al. 407* (COL); 15 Jan 2007, st., *A. Aldana et al. 406* (COL); Magdalena Valley. 30 Sep 1977, st., *A. Gentry & E. Renteria 20065* (COL, MO); 30 Sep 1977, fr., *A. Gentry & E. Renteria 20106* (MO); Santander. 2 Sep 1954, st., *R.R. Castañeda 4813* (COL); Cimitarra. 21 Sep 2005, st., *Z. Cordero-P et al. 907* (COAH, COL); 11 Oct 2001, st., *J.G. Vélez et al. 4563* (HUA); Vélez, 22 Aug 1966, fr., *L. Vega & H. Sánchez 20* (COL).

*Common names: arenillo* (Spanish).

*Distribution and Habitat:* Restricted to the inter-Andean region of Colombia on well-drained, sandy-clay soils in areas with steep slopes at elevations of 200–700 m.s.m. (Figure 6).

*Phenology:* Flowers are unknown; fruiting specimens were collected from January to September.

*Conservation Status:* Although with restricted distribution, the EOO is greater than 20,000 km^2^ and at least one specimen is registered in a protected region (Reserva Natural de Aves El Paujil). Therefore, *C. digitatum* is herein tentatively considered LC.

*Notes:* Distinct from the other species by the digitate leaves in adult individuals, resembling only *C. commune*. It differs from that species by the leaves, usually (3–)5(–7)-foliolate on all branches (vs. sometimes trifoliolate, and usually on branches also with unifoliolate leaves). When realized to be a new species in 1976–1977, it was first considered by the authors, and Alwyn Gentry, as a potentially new genus of “Bombacaceae” (now Bombacoideae, Malvaceae) for its combination of digitately compound adult leaves and single-seeded fruits.

***Catostemma durifolium*** W.S. Alverson, Novon 4(1): 3. 1994. Type: VENEZUELA. Bolívar. along Río Sarven between camps 3 and 4, slopes and talus forest. Sarvén-tepuí, 1400 m, 10 Jan 1953, *Wurdack 34085* (holotype: WIS!; isotypes: MO!; NY [2 sheets]!; VEN [photo]!).

Figure 4G

Trees 12–25 m tall. Branches not glaucous, densely covered with brown and hyaline, fasciculate trichomes. Stipules ca. 0.6 × 0.8 cm, apices acute, both surfaces not glaucous, the adaxial surface glabrous, the abaxial surface densely covered with brown, fasciculate trichomes. Seedling leaves not seen. Leaves of adult trees unifoliolate, petioles 2.1–5.7 cm long, not glaucous, densely covered with brown and hyaline, fasciculate trichomes, leaflets 21.5–25.6(–43) × 8.9–11.7(–22) cm, coriaceous, bullate, elliptic to oblong, margins revolute, bases rounded or subcordate, apices rounded and mucronate, the adaxial surface glabrous or with veins sparsely covered with hyaline, fasciculate trichomes, the abaxial surface densely covered with brown, fasciculate trichomes and without evident stomata, tertiary veins mixed percurrent. Inflorescences 4–6-fasciculate, axillary, 3-bracteolate; bracteoles ca. 0.5 × 0.3 cm, triangular, apices acuminate, their adaxial surface glabrous and abaxial surface sparsely covered with brown, fasciculate trichomes. Floral pedicels 8.1–9.6 cm long, densely covered with brown, fasciculate trichomes; hypanthia 0.3–0.5 × 0.9–1 cm, the adaxial surface glabrous; calyx tubes 0.6–0.8 × 1.4–1.5 cm, the adaxial surface glabrous, apices 2–3-lobate, lobes 1.4–1.5 × 1–1.4 cm, lobe apices obtuse, the adaxial surface sparsely covered with fasciculate and simple hyaline trichomes; abaxial surfaces of the hypanthium, calyx tube, and calyx lobes with the same indument as the pedicel; petals 2.9–3.5 × 1.6–1.8 cm, oblanceolate, apices rounded and their adaxial surfaces sparsely covered with hyaline, fasciculate trichomes, the abaxial surface densely covered with brown, fasciculate trichomes; staminal columns ca. 0.5 × 0.7–0.8 cm, free filaments 450–500, 1.6–2.1 cm long, glabrous; ovaries 0.5–0.6 × 0.5–0.6 cm, 4-locular with 3 ovules per locule; styles ca. 1.6 cm long, the basal and median portions densely covered with brown, fasciculate trichomes; stigmas 4-fid. Immature fruits ca. 10–13 × 6–8 cm, ellipsoid, 1(–2)-seeded; immature seeds 4–5 × 2–3 cm fusiform or ovoid.

*Specimens Examined:* BRAZIL. Roraima. Parque Nacional do Monte Roraima [Cuyuni-Mazaruni Region], 14 June 2004, fl., *H.D. Clarke et al. 11645* (NY); GUYANA. Cuyuni-Mazaruni Region. 22 May 2012, fl., *K.M. Redden et al. 7286* (NY); Potaro-Siparuni Region. 26 Oct 1994, fr., *P. Mutchnick et al. 235* (US); VENEZUELA. Bolivar. Gran Sabana. 23 Mar 1952, st., *B. Maguire 33541* (NY); 29 Apr 1987, fl., *L. Hernández 500* (MO); 3 May 1987, fr., *L. Hernández 520* (MO); Sierra Parima., 8 Apr 1946, st., *F. Cardona 1491* (US).

*Common names: baro-malle, chimanayek, paloo* (unknown).

*Distribution and Habitat:* Occurs in Roraima state in Brazil, Cuyuni-Mazaruni in Guyana, and the state of Bolivar in Venezuela, on well-drained sandy soils in areas with steep slopes at elevations of 450–1530 m.s.m. (Figure 6).

*Phenology:* Flowering specimens were collected from April to June and fruiting specimens from May to October.

*Conservation Status:* Restricted to high altitude regions, its EOO is greater than 20,000 km^2^, and at least one specimen is registered in a protected region (Parque Nacional do Monte Roraima), so *C. durifolium* is herein considered LC.

*Notes:* This differs from other species by its large leaflets (21.5–25.6(–33.1) × 8.9–11.7(–17.1) cm) that are conspicuously bullate and often very firm; also by its long pedicels (8.1–9.6 cm) and free filaments (1.6–2.1 cm). It is the only species of the clade with a 4-locular ovary.

***Catostemma ebracteolatum*** Steyerm., Ann. Missouri Bot. Gard. 74: 641 1987. Type: VENEZUELA. Amazonas. Cerro Sipapo (Paráque), watercourse above Intermediate Camp, 2 Feb 1949, *B. Maguire & L. Politi 28741* (holotype: MO [2 sheets] [photos]!; isotypes: MG [photo]!; NY [photo]!; US [photo]!; VEN [photo]!).

*Catostemma hirsutulum* Steyerm., Ann. Missouri Bot. Gard. 74: 642. 1987. Type: VENEZUELA. Bolívar. Chimantá Massif, rich rain forest on northwestern slopes of Abácapa-tepui, vicinity of camp 1 along Río Abácapa, 30–31 Mar 1953, *J.A. Steyermark 74781* (holotype: MO [photo]!; isotypes: BM [photo]!; F, not seen; NY [photo]!; VEN [photo]!) **Syn. nov.**

Figure 4E,P

Trees 15–30 m tall. Branches usually glaucous, glabrous or densely to sparsely covered with hyaline or brown, fasciculate trichomes. Stipules 0.4–0.6 × 0.2–0.4 cm, apices attenuate to caudate, both surfaces not glaucous and densely covered with brown and/or nigrescent glandular trichomes, sometimes with an abaxial surface at apex sparsely covered with hyaline, fasciculate trichomes. Seedling leaves not seen. Leaves of adult trees unifoliolate, petioles 0.4–1.8(–2.3) cm long, usually glaucous, glabrous or densely to sparsely covered with hyaline or brown fasciculate trichomes, leaflets 3.4–6.5 × 1.5–3.5 cm, coriaceous, not bullate, obovate to oblanceolate, margins entire, not revolute, bases cuneate or truncate, apices retuse to emarginate and mucronate, both surfaces densely covered with brown and/or nigrescent, glandular trichomes, without evident stomata, the tertiary veins alternate percurrent. Inflorescences 2–3-fasciculate, axillary, ebracteolate. Floral pedicels 1.4–1.9 cm long, densely covered with brown, short fasciculate trichomes, and long hyaline fasciculate trichomes, both arranged in longitudinal rows; hypanthia 0.2–0.3 × 0.4–0.5 cm; calyx tubes ca. 0.1 × 0.3–0.4 cm, apices 2-lobate, lobes 0.7–0.9 × 0.9–1.1 cm, lobe apices rounded, sparsely covered with hyaline, simple, and fasciculate trichomes, the adaxial surfaces of the hypanthium and calyx tube glabrous, abaxial surfaces of the hypanthium and calyx with same indument of the pedicel; petals 1.1–1.5 × 0.5–0.6 cm, oblong, apices rounded, the adaxial surface glabrous, the apex of the adaxial surface sparsely covered with hyaline, fasciculate trichomes; staminal columns 0.15–0.2 × 0.3–0.35 cm, free filaments 100–130, 0.7–0.9 cm long, glabrous; ovaries ca. 0.3 × 0.2 cm, 2-locular with 2 ovules per locule; styles 1–1.4 cm long, glabrous; stigmas bifid. Immature fruit s6.3–8.3 × 2.9–3.8 cm, obovoid to fusiform, 1-seeded; immature seeds 4.2–5.4 × 2.3–2.4 cm, obloid.

*Specimens Examined:* VENEZUELA. Amazonas. Atabapo. Mar 1992, st., *L. Delgado 1679* (NY); Atures. 13 May 1980, st., *J.A. Steyermark et al. 122402* (MO); Bolivar. Caño Pablo. 5 July 1982, st., *G. Morillo & R. Liesner 8944* (MO, NY); Cedeño. Mar 1989, fl. and fr., *Y. Fernandez & M. Yanez 377* (MO, NY); Apr 1989, fr., *A. Fernandez 5287* (MO, NY); El Cacaro. May 1989, fr., *E. Marin 224* (MO); Piar. 2 May 1986, fr., *B. Holst et al. 2759* (MO, NY); Raul Leoni. Apr 1988, fr., *A. Fernandez 4374* (MO, NY, VEN); San Carlos de Rio Negro. 12 June 1981, fr., *H.L. Clark & P. Maquirino 8081* (COL, MO, NY, QCNE); Sierra Ichún. 28 Dec 1961, fl. and fr., *J.A. Steyermark 90371* (NY); Sucre. June 1989, fr., *Y. Fernandez & M. Yanez 447* (NY, QCNE, VEN).

*Common names: baraman* (Spanish); *coomayo*, *kömayu* (Yekuana); *guekupiyek* (Pemón); *tetón* (likely Spanish).

*Distribution and Habitat:* Occurs widely in Venezuela on poorly drained, sandy soils sometimes subject to seasonal flooding, at elevations of 100–500 m.s.m. (Figure 7).

*Phenology:* Flowering specimens were collected from December to March and fruiting specimens from December to June.

*Conservation Status:* A widely distributed species with an EOO greater than 20,000 km^2^ and several known occurrence localities, *C. ebracteolatum* is herein considered LC.

*Notes:* Morphologically like *C. commune* and *C. grazielae*, the main similarities and characteristics that distinguish the three species are addressed in the comments here under *C. commune*.

Steyermark [7] published *C. ebracteolatum* and *C. hirsutulum* based on specimens collected in Venezuela, in Amazonas and Bolivar states, respectively. While *C. ebractolatum* was described from specimens with flowers, *C. hirsutulum* was published with only a description of its fruits. Vegetatively, the two species are morphologically similar in the size and shape of their leaflets, differing only in the indument of the branches and petioles: glabrous in *C. ebracteolatum* and pilose in *C. hirsutulum*. However, at least one specimen that we analyzed (*E. Marin 224*) presents both glabrous and pilose petioles on the same branch, as such an intermediate condition between the two taxa. The presence or absence of trichomes on these structures is variable and probably influenced by both branch age and environmental factors. For these reasons, here we propose *C. hirsutulum* as a synonym of *C. ebracteolatum*.

***Catostemma fragrans*** Benth., London J. Bot. 2: 365. 1843. Type: GUYANA. Banks of rivers, 1837, *R. Schomburgk 280* (lectotype, first-step designated by Steyermark [7], second-step by Ferreira et al. [17]: K [photo]!; isolectotypes: BM [photo]!; K [photo]!; L barcode L0424693 [photo]!; P [photo]!; US [photo]!; W [photo]!).

*Catostemma macrospermum* (Sagot ex Benoist) Lemée, Fl. Guyane Franç. 3: 631. 1954. *Guenetia macrosperma* Sagot ex Benoist, Bull. Mus. Hist. Nat. (Paris) 25: 387. 1919. Type: FRENCH GUIANA. Acarouany. 1855, *Sagot s.n.* (lectotype, first-step designated by Steyermark [7], second-step by Ferreira et al. [17]: P [photo]!; isolectotype: P [photo]!).

Figure 4H

Trees 25–35 m tall. Branches usually glaucous. glabrous or densely covered with hyaline, fasciculate trichomes. Stipules 0.5–0.9 × ca. 0.3 cm, apices attenuate, both surfaces not glaucous, and densely covered with brown or hyaline, fasciculate trichomes. Seedling leaves unifoliolate, petioles (3.8–)4.8–7.4 cm long, glabrous or sparsely covered with hyaline, fasciculate trichomes, usually glaucous, leaflets 13.1–29.5 × 4.7–8 cm, membranaceous to chartaceous, not bullate, oblanceolate or rarely elliptic, margins entire, not revolute, bases rounded, apices caudate and mucronate, the adaxial surface glabrous or with a midvein sparsely covered with brown, fasciculate trichomes, the abaxial surface densely covered with brown, glandular trichomes, its veins sparsely covered with brown, fasciculate trichomes, without evident stomata, the tertiary veins mixed percurrent. Leaves of adult trees unifoliolate, petioles 1.2–5.3 cm long, not glaucous, densely covered with brown, fasciculate trichomes, leaflets (6.4–)8.8–23.5 × (3.3–)3.8–8.9 cm, chartaceous to coriaceous, not bullate, obovate to oblanceolate, rarely elliptic, margins entire, rarely slightly revolute, bases rounded, apices retuse or emarginate, mucronate, the adaxial surface with same indument as the seedling leaves, the abaxial surface sparsely or densely covered with brown, fasciculate and glandular trichomes, the veins sparsely or densely covered with brown and hyaline, fasciculate and stellate-rotate trichomes, without evident stomata, the tertiary veins mixed percurrent. Inflorescences (3–)5–9-fasciculate, axillary, 3-bracteolate; bracteoles 0.1–0.2 × 0.1–0.2 cm, triangular, apices obtuse or acute, the adaxial surface glabrous, the basal and mid-portions of the abaxial surface densely covered with brown, fasciculate trichomes, apices glabrous. Floral pedicels 2.2–3.8 cm long, densely covered with brown, fasciculate trichomes; hypanthia 0.1–0.2 × 0.4–0.5 cm, their adaxial surface densely covered with brown, fasciculate trichomes arranged in longitudinal rows; calyx tubes 0.1–0.2 × 0.6–0.7 cm, the adaxial surface glabrous, apices 2-lobate, lobes 0.8–0.9 × 0.5–0.7 cm, lobe apices rounded to obtuse, the adaxial surfaces glabrous, the abaxial surfaces of the hypanthium, calyx tube, and calyx lobes with the same indument as the pedicel; petals 1.6–1.8 × 0.4–0.7 cm, oblong or slightly obovate, apices obtuse, the adaxial surface glabrous or sparsely covered with hyaline, fasciculate trichomes, the abaxial surface sparsely covered with hyaline, fasciculate trichomes; staminal columns ca. 0.2 × 0.3 cm, free filaments 80–120, 0.7–0.9 cm long, innermost filaments densely covered with brown, fasciculate trichomes; ovaries ca. 0.5 × 0.3 cm, 3-locular with 2 ovules per locule; styles 1.2–1.5 cm long, glabrous or densely/sparsely covered with brown, fasciculate trichomes; stigmas trifid. Fruits 6–8.9 × 3–3.5 cm, fusiform to ellipsoid, 1(–2)-seeded; seeds 3.9–5.2 × 2.1–2.7 cm, fusiform to ellipsoid.

*Representative Specimens Examined:* FRENCH GUIANA. 13 July 1921, st., *G. Wachenheim s.n.* (NY); 23 Mar 1990, fl., *D. Sabatier 3046* (NY); 21 July 1986, st., *D. Sabatier & M.F. Prèvost 1331* (NY); Awala-Yalimapo. 20 Nov 2006, seed., *P. Grenand et al. 3381* (MO, NY); Kourou River. 20 Sep 1967, fr., *B. Oldeman B-1351* (NY); Sinnamary River. 26 Apr 1968, seed., *B. Oldeman 1600* (NY); 2 Sep 1993, st., *S. Mori et al. 23534* (NY); GUYANA. Aravina. 3 July 1991, fr., *S. Tiwari & A. Mengharini 176* (NY); Barima River. 19–22 Mar 1923, fl., *J.S. Cruz 3348* (MO, NY, VEN); Essequibo River. 15–24 Dec 1937, fr., *A.C. Smith 2739* (MO, NY); 19 Oct 1929, seed., *N.Y. Sandwith 485* (NY, RB); 27 Sep 1989, fr., *M.J. Jansen-Jacobs et al. 1855* (NY); 2 Oct 1989, fr., *M.J. Jansen-Jacobs et al. 1929* (MG, MO, NY); 25 Sep 1993, fr., *T.W. Henkel et al. 3172* (MO, NY); 19 Feb 1997, fr., *H.D. Clarke 3670* (NY); 3 May 1996, fr., *H.D. Clarke & T. McPherson 1823* (MO, NY); 21 Sep 1999, fr., *H.D. Clarke et al. 8843* (NY); 15 Sep 1999, fr., *H.D. Clarke et al. 8621* (MO, NY); 20 Sep 1999, fr., *M.J. Jansen-Jacobs 6038* (MO, NY); Nov 1996, fr., *H.D. Clarke 3262* (MO); Iwokrama Reserve, 18 Feb 1995, fr., *P. Mutchnick 802* (NY); Himara Creek. 16 Sep 2008, fr., *K.M. Redden et al. 6036* (NY); Mabura Hill. 12 Oct 1989, fr., *M.J. Jansen-Jacobs et al. 1979* (NY); Potaro-Siparuni. Kaieteur National Park, 11–19 Mar 1997, st., *C.L. Kelloff et al. 1256* (MO); Seballi. 2 Dec 1991, st., *M. Polak 573* (NY); SURINAME. Lucie Rivier. 9 July 1963, fl., *B. Maguire et al. 54098* (MO, NY, RB); Maratakka River. 1 May 1965, fr., *P.J. Maas 10799* (NY); National Park Raleigh falls, 12 Feb 1975, fl., *L. Roberts 14764* (NY); Sipaliwini. 26 Nov 1994, fr., *R. Evans et al. 2003* (MO); 3°54’30”N 56°10’35”W, 6 July 2001, st., *R. Evans 3315A* (MO); 15 Aug 2013, fl., *F.A. Michelangeli & J. Aguirre-Santoro 2075* (NY); Tafelberg, 30 July 1944, fl., *B. Maguire 25069* (MO, NY). See additional specimens examined in Appendix A.

*Common names: aganananga* (Paramaka); baromalli-of-Wallaba, sand-baromalli (English); *kulebogo* (Kali’na).

*Distribution and Habitat:* Occurs widely in French Guiana, Guyana, and Suriname on poorly drained, sandy soils subject to seasonal flooding, at elevations of 10–500 m.s.m. (Figure 7).

*Phenology:* Flowering specimens were collected from February to August and fruiting specimens from February to December.

*Conservation Status:* A widely distributed species with an EOO greater than 20,000 km^2^, several known occurrence localities, and specimens registered in protected regions (Iwokrama Reserve, Kaieteur National Park, National Park Raleigh falls), *C. fragrans* is herein considered LC.

*Notes:* This differs from the other species of *Catostemma* by the indumentum of the abaxial face of the leaflets of adult individuals, which are covered by fasciculate and glandular trichomes, and by its veins, which are covered with fasciculate and stellate-rotate trichomes. Also, the indumentum of the adaxial face of the hypanthium is covered by fasciculate trichomes organized in longitudinal rows, and free innermost filaments have the same indumentum.

***Catostemma grazielae*** Paula, Ci. & Cult. 21(4): 703. 1969. Type: BRAZIL. Amapá. rio Oiapoque, primary forest along road between Oiapoque and Clevelândia, 20 July 1960, *B. Maguire et al. 47082* (holotype: IAN [photo]!; isotype: MG [photo!]; NY [photo]!; U [photo]!; UB [photo]!; US [photo]!).

Figure 4I

Trees ca. 40 m tall. Branches usually glaucous, glabrous. Stipules ca. 0.5 × 0.2 cm, apices attenuate, both surfaces not glaucous, and densely or sparsely covered with brown, glandular trichomes, sometimes only at apex. Seedling leaves not seen. Leaves of adult trees unifoliolate, petioles (2–)4.1–4.9 cm long, not glaucous, glabrous, leaflets 8.4–14 × 3.9–7.5 cm, coriaceous, not bullate, obovate, margins entire, not revolute, bases obtuse to cuneate, apices retuse to emarginate and mucronate, both surfaces densely covered with brown and/or nigrescent, glandular trichomes, without evident stomata, the tertiary veins alternate percurrent. Inflorescences 3–5-fasciculate, axillary, 3-bracteolate; bracteoles ca. 0.2 × 0.2 cm, triangular, apices acute, both surfaces densely covered with brown, fasciculate trichomes. Floral pedicels 0.4–0.7(–1.1) cm long, densely with brown, fasciculate trichomes; hypanthia 0.1–0.2 × 0.3–0.5 cm; calyx tubes 0.1–0.2 × 0.5–0.7 cm, apices 3-lobate, lobes 0.4–0.7 × 0.3–0.5 cm, lobe apices acute to obtuse, the adaxial surfaces of the hypanthium and calyx tube glabrous, apices of the calyx lobes densely covered with hyaline, fasciculate trichomes; abaxial surfaces of the hypanthium and calyx with same indument of the pedicel; petals 0.7–0.9 × 0.3–0.4 cm, oblong, apices rounded to obtuse and both surfaces sparsely covered with hyaline, fasciculate trichomes; staminal columns 0.2–0.3 × 0.5–0.6 cm, free filaments 90–120, 0.9–1.1 cm long, glabrous; ovaries 0.3–0.4 × 0.2–0.3 cm, 3-locular with 2 ovules per locule; styles ca. 1.2 cm long, glabrous; stigmas trifid. Immature fruits 7.5–9 × 3–4 cm, fusiform to slightly ovoid, 1-seeded; immature seeds ca. 4.7 × 1.5 cm, obloid.

*Specimens Examined:* BRAZIL. Amapá. Oiapoque. 5 Dec 1984, fr., *S. Mori et al. 17191* (MG, MO, US).

*Common names*: Unknown.

*Distribution and Habitat:* Endemic to Oiapoque, Amapá, Brazil, where it grows on well-drained clay soils not subject to seasonal flooding, at elevations of 50–80 m.s.m. (Figure 7).

*Phenology:* Flowering specimens were collected in July and fruiting specimens in December.

*Conservation Status:* This species has a restricted distribution, not exceeding 10 km^2^ of AOO, is known from only one locality, not included in any protected area, and was last collected in 1984; *C. grazielae* is herein considered DD. Expeditions to locate this species in the field were not possible during our study. Future efforts are necessary to understand the conservation status of this species better.

*Notes:* Morphologically like *C. commune* and *C. ebracteolatum*, the main similarities and characteristics that distinguish the three species were addressed in the *C. commune* comments.

***Catostemma lanceolatum*** C.D.M.Ferreira & W.S.Alverson Phytotaxa 606(4): 296. 2023. Type: BRAZIL. Amazonas. Presidente Figueiredo [São Sebastião do Uatumã], Reserva de Desenvolvimento Sustentável do Uatumã, Rio Abacate. 10 May 2017, fl., *L.O. Demarchi & L.E. Barcelos 872* (holotype: RB!, isotype: INPA!).

Figure 4A,N,O

Trees 15–30 m tall. Branches usually glaucous, glabrous. Stipules ca. 0.5 × 0.4 cm, apices cuspidate, both surfaces not glaucous, the adaxial surface glabrous, the abaxial surface densely to sparsely covered with brown or hyaline, fasciculate trichomes. Seedling leaves not seen. Leaves of adult trees unifoliolate, petioles (2–)3.5–4.3(–9.6) cm long, rarely glaucous, glabrous, leaflets 8.9–15.4(–23.9) × 3.1–4.4(–10.4) cm, chartaceous to coriaceous, not bullate, lanceolate to narrowly ovate, margins entire, not revolute, base rounded or attenuate, apices obtuse and mucronate, both surfaces glabrous, without evident stomata, the tertiary veins alternate percurrent. Inflorescences 3–9-fasciculate, axillary, 3-bracteolate; bracteoles 0.3–0.8 × 0.2–0.4 cm, filiform, apices acuminate, the adaxial surface of the midvein densely covered with simple, hyaline trichomes, the abaxial surface densely covered with brown, fasciculate trichomes. Floral pedicels 7.1–8.4 cm long, densely covered with brown, fasciculate trichomes; hypanthia ca. 0.3 × 0.6 cm, their adaxial surface glabrous; calyx tubes ca. 0.2 × 0.8 cm, its adaxial surface glabrous or sparsely covered with simple, hyaline trichomes, apices 3-lobate, lobes ca. 1.5 × 0.6–1.1 cm, lobe apices acute, adaxial surface of the calyx lobes densely covered with hyaline, simple trichomes; the abaxial surfaces of the hypanthium, calyx tube, and calyx lobes with same indument as the pedicel; petals 2.1–2.5 × 1.2–1.7 cm, obovate, apices rounded, the adaxial surface sparsely covered with hyaline, simple trichomes, the abaxial surface densely covered with similar trichomes, the basal portion glabrous; staminal columns 0.2–0.3 × 0.3–0.4 cm, free filaments 420–450, ca. 1.8 cm long, glabrous; ovaries ca. 0.3 × 0.25 cm, 3-locular with 2 ovules per locule; styles ca. 1.6 cm long, glabrous; stigmas trifid. Immature fruits ca. 10 × 2.4 cm, fusiform or obloid; seeds not seen.

*Specimens Examined:* BRAZIL. São Sebastião do Uatumã. Balbina. 18 Feb 1988, fl., *A. Knob et al. 791* (HUAM); st., *192* (HUAM); 5 Aug 2006, fr., *C.E. Zartman et al. 5875* (INPA); 23 Mar 1983, st., *F.R. Silva 200* (INPA, RB); 23 Mar 1983, fr., *F.R. Silva 203* (INPA, RB); 23 Mar 1983, fr., *F.R. Silva 204* (INPA, RB); 25 July 1988, fl., *V. Carvalho et al. 78* (HUAM).

*Common names*: Unknown.

*Distribution and Habitat:* Endemic species of the municipality of São Sebastião do Uatumã, Amazonas, Brazil, which occurs on poorly drained, sandy soils subject to seasonal flooding, at elevations of 10–20 m.s.m. (Figure 8).

*Phenology:* Flowering specimens were collected in May and fruiting specimens in March.

*Conservation Status:* This species has a restricted distribution, not exceeding 5000 km^2^ of EOO, and is known from only two localities, with only one protected (Reserva de Desenvolvimento Sustentável do Uatumã). Field data collected by Ferreira et al. [13] indicate that this species frequently occurs in the *igapós* (Amazonian floodplain forest permanently inundated, with vegetation adapted to anoxic, nutrient-poor soils) of the São Sebastião do Uatumã region, but current herbarium specimens are insufficient to reliably confirm the distribution and the actual range of this species. Additional records are needed to better document the full distribution of this species, so *C. lanceolatum* is herein considered DD, as in the previous analysis by Ferreira et al. [13].

*Notes:* This differs from other species of *Catostemma* by its lanceolate to narrowly ovate leaflets and long (0.3–0.8 cm), filiform bracteoles. Specimens of this species were recognized by its longer pedicels (7.1–8.4 cm vs. 3.4–5 cm), larger hypanthium (ca. 0.3 × 0.6 cm vs. 0.1–0.2 × 0.3 cm), larger size and shape of the calyx-lobe apices (ca. 1.5 × 0.6–1.1 cm with acute apices vs. 0.7–0.8 × 0.5–0.6 cm with obtuse apices), larger petals (2.1–2.5 × 1.2–1.7 cm vs. ca. 1.2 × 0.5–0.6 cm), and greater quantity and length of filaments (420–450, ca. 1.8 cm long vs. 120–140, 0.6–0.7 cm long).

***Catostemma milanezii*** Paula, Ci. & Cult. 21(4): 702. 1969. Type: BRAZIL. Amazonas. Manaus, Reserva Florestal Ducke, mata de terra firme, 27 June 1967, fr., *J. Elias 401* (holotype: INPA [2 sheets] [photo]!). Brazil. Amazonas, Manaus, BR-174, km 27, num varadouro do lado direito. Mata de Terra Firme, 10 Mar 1970, fl., *W.A. Rodrigues 8757* (epitype, designated by Ferreira et al. [17]: RB [2 sheets]!; isoepitype: INPA!).

Figure 4J

Trees 20–35 m tall. Branches usually glaucous and glabrous. Stipules ca. 0.4 × 0.2 cm, apices attenuate, both surfaces not glaucous and densely covered with brown, fasciculate trichomes. Seedling leaves not seen. Leaves of adult trees unifoliolate, petioles (1.3–)2.2–4.3 cm long, not glaucous, glabrous, or the pulvinus sparsely covered with brown, fasciculate trichomes, leaflets 8.2–16.7 × 3.6–6.2 cm, chartaceous, not bullate, elliptic to obovate, margins entire, not revolute, bases rounded or slightly attenuate, apices acuminate, mucronate, the adaxial surface glabrous, the abaxial surface densely covered, bearing conspicuous stomata and veins with sparsely covered brown, fasciculate trichomes, the tertiary veins mixed percurrent. Inflorescences 2–3-fasciculate, axillary, 3-bracteolate; bracteoles ca. 0.2 × 0.1 cm, triangular, apices cuspidate, the adaxial surface glabrous, the abaxial surface densely covered with brown, fasciculate trichomes, the basal portion glabrous. Floral pedicels (0.7–)1.1–1.7 cm long, densely with brown, fasciculate trichomes; hypanthia ca. 0.1 × 0.3 cm; calyx tubes ca. 0.1 × 0.4 cm, apices 2-lobate, lobes 0.5–0.6 × 0.5–0.6 cm, lobe apices acuminate, the adaxial surface of the hypanthium and calyx glabrous, the abaxial surfaces of the hypanthium, calyx tube, and calyx lobes, with same indument as the pedicel; petals ca. 1.1 × 0.3–0.4 cm, oblong, apices rounded and both surfaces sparsely covered with hyaline, fasciculate trichomes; staminal columns ca. 0.1 × 0.2 cm, free filaments 110–140, 0.6–0.7 cm long, glabrous; ovaries ca. 0.3 × 0.2 cm, 3-locular, with 2 ovules per locule; styles 0.8–1 cm long, glabrous; stigma trifid. Immature fruits 8.9–11.3 × 2.6–3.5 cm, fusiform, 1-seeded; immature seeds 6.4–6.8 × 2.5–3.1 cm, obloid.

*Specimens Examined:* BRAZIL. Amazonas. Manaus. Reserva Florestal Adolpho Ducke, 7 Nov 2021, st., *C.D.M. Ferreira et al. 859* (RB); 12 Apr 1994, fr., *J.R. Nascimento et al. 502* (K, MG, MO, NY, RB, SP); 31 July 1997, fr., *G.L. Esteves et al. 7/97* (INPA, RB); 5 May 1967, fr., *J.E. Paula 396* (INPA, RB); 5 Aug 1994, fr., *M.J.G. Hopkins et al. 1478* (IAN, INPA, SP, UB).

*Common names: falso-cardeiro* (Portuguese).

*Distribution and Habitat:* An endemic species of the municipality of Manaus, Amazonas, Brazil that occurs on well-drained clay soils not subject to flooding, at elevations of 100–200 m.s.m. (Figure 8).

*Phenology:* Flowering specimens were collected in March and fruiting specimens from April to August.

*Conservation Status:* This species has a restricted distribution, and is known from a single locality, the protected area Reserva Florestal Adolfo Ducke. All collections of this species represent only a single known individual. Therefore, population data and reliable information on the actual distribution of this species are lacking. While additional populations of this species might exist, we currently know of only a single individual. Even with our dedicated efforts during this project, we could not find another individual of this taxon. For these reasons, *C. milanezii* is herein considered CR (D1).

*Notes:* Similar to *C. durifolium*, *C. albuqueruei*, and *C. fragrans* in those tertiary veins are opposite and alternate percurrent, this species can be distinguished by several features. The adult leaflets of *C. durifolium* are bullate (a trait not observed in *C. milanezii*) and differ in the size (21.5–25.6[–33.1] × 8.9–11.7[–17.1] cm), margins (revolute), indumentum of the abaxial surface (densely covered with brown, fasciculate trichomes), and consistency (chartaceous or indurate). It can be differentiated from *C. fragrans* by the indumentum of adult leaflets: in *C. fragrans*, the adaxial surface is glabrous or the midvein is sparsely covered with brown, fasciculate trichomes, the abaxial surface is sparsely or densely covered with brown, fasciculate and glandular trichomes, and the veins are sparsely or densely covered with brown and hyaline, fasciculate and stellate-rotate trichomes. It also can be distinguished from *C. albuquerquei* by the shape, margin, and indumentum of adult leaflets. In *C. albuquerquei*, leaflets are oblong or rarely slightly ovate, with a revolute margin, and the abaxial surface is densely covered with brown, fasciculate trichomes. Regarding floral characteristics, *C. milanezii* differs from other species by having 2-lobed calyx with an acuminate apex and a glabrous adaxial surface.

***Catostemma sclerophyllum*** Ducke, Trop. Woods 50: 39. 1937. Type: BRAZIL. Amazonas. Manaus, Estr. do Aleixo [currently Av. André Araújo], km 12, 29 May 1936, fl., *A. Ducke s.n.* (lectotype, designated by Ferreira et al. [17]: RB!; isolectotypes: K [photo]!; MBM [photo]!; MG [photo]!; NY [photo]!; NY [photo]!; NY [photo]!; P [photo]!; RB [fragment]!; RB!; S [photo]!; U *p.p.* [branch with flowers in anthesis, and sterile branch] [photo]!; UFMT!; US [photo]!; WIS [photo]!).

Figure 4K,L

Trees 10–15 m tall. Branches glaucous, glabrous. Stipules ca. 0.2 × 0.3 cm, apices acuminate, both surfaces not glaucous and densely or sparsely covered with brown, glandular trichomes. Seedling leaves not seen. Leaves of adult trees unifoliolate, petioles (1.6–)2.6–8.7 cm long, glaucous, glabrous, leaflets 7.7–17.1 × 4.2–10.8 cm, coriaceous, not bullate, oblong or obovate to lanceolate, margins entire, not revolute, bases rounded or subcordate to cordate, apices emarginate or obtuse to attenuate and mucronate, both surfaces glabrous and with dense, conspicuous stomata, the tertiary veins alternate percurrent. Inflorescences 5–14-fasciculate, axillary, 3-bracteolate; bracteoles, ca. 0.1 × 0.1 cm, triangular, apices obtuse or cuneate, both surfaces densely covered with brown, fasciculate trichomes. Floral pedicels 3.4–5 cm long, densely covered with brown, fasciculate trichomes; hypanthia 0.1–0.2 × 0.3 cm; calyx tubes ca. 0.2 × 0.4 cm, apices 3-lobate, lobes 0.7–0.8 × 0.5–0.6 cm, lobe apices obtuse, adaxial surface of the hypanthium, calyx tube, and calyx lobes, glabrous; abaxial surfaces of the hypanthium, calyx tube, and calyx lobes with same indument as the pedicel; petals ca. 1.2 × 0.5–0.6 cm, obovate, apices rounded and both surfaces densely covered with hyaline, simple trichomes; staminal columns ca. 0.2 × 0.2 cm, free filaments 120–140, 0.6–0.7 cm long, glabrous; ovaries 0.3–0.4 × ca. 0.3 cm, 3-locular with 2 ovules per locule; styles 1.1–1.4 cm long, glabrous; stigmas trifid. Fruits ca. 10 × 4.3 cm, fusiform, 1(–2)-seeded; immature seeds 4.4–4.9 × 3.1–3.4 cm, ellipsoid.

*Specimens Examined:* BRAZIL. Amazonas. Manaus. Av. André Araújo, 29 May 1948, fl., *T. Guedes 43* (IAN); 6 May 1942, fl., *A. Ducke s.n.* (MG, MO, US); Igarapé do Buião. May 1971, fl., *D.F. Coêlho & O.P. Monteiro s.n.* (EAFM, INPA, MO, RB); Pensador. 18 Sep 1941, fr., *A. Ducke s.n.* (IAN, US); Reserva Florestal Adolpho Ducke, 13 May 1997, fr., *C.A. Sothers & P.A.C.L. Assunção 979* (INPA, RB); 13 Sep 1995, fr., *J.E.L.S. Ribeiro & E.C. Pereira 1706* (MO, RB); 29 Oct 2021, st., *C.D.M. Ferreira et al. 831* (RB); Rio Tarumá. 1 May 1942, fl., *A. Ducke 1469* (IAN, MG, RB, US); 27 June 1941, fr., *A. Ducke s.n.* (MG, RB); Presidente Figueiredo. 2 Aug 1993, fr., *C.A.C. Ferreira & J.F. Ramos 11028* (INPA, RB), Reserva de Desenvolvimento Sustentável do Uatumã, 3 Oct 2018, fr., *L.O. Demarchi 1385* (INPA, RB); Roraima. BR-174, km 350, 18 Nov 1977, fr., *W.C. Steward et al. 90* (US); 22 Aug 1987, fr., *C.A. Cid Ferreira 9114* (US); 23 Aug 1987, fl. and fr., *C.A. Cid Ferreira 9142* (INPA, MG, US); 15 Mar 1984, fr., *J.L. dos Santos & J.A. Coêlho 676* (INPA, MO, RB); Rio Branco. Rio Anauá, 4 Mar 1978, fr., *N.T. Silva 4528* (MG).

*Common names: folha-de-flandre* (Portuguese).

*Distribution and Habitat:* Occurs in Amazonas and Roraima states in Brazil on poorly drained, sandy soils, usually close to *Igarapés* but in places not subject to seasonal flooding, at elevations of 40–80 m.s.m. (Figure 8).

*Phenology:* Flowering specimens were collected from May to August and fruiting specimens from July to March.

*Conservation Status:* Although it has a restricted distribution, its EOO is greater than 20,000 km^2^ and some specimens recorded in protected regions (Reserva de Desenvolvimento Sustentável do Uatumã, Reserva Florestal Adolpho Ducke). Therefore, *C. sclerophyllum* is herein considered LC.

*Notes:* This differs from the other species by its oblong leaflets (specimens from Amazonas) or obovate to lanceolate leaflets with a subcordate to cordate base (specimens from Roraima). Although *C. sclerophyllum* does not share many similarities with *C. lanceolatum*, they are commonly confused in herbaria. This likely occurs due to the similarity and proximity of their habitats. Both species occur in sandy, poorly drained soils at low elevations (10–80 m.s.m.) in the municipality of São Sebastião do Uatumã, within the Uatumã Sustainable Development Reserve. The differences between the two species are described in the comments on *C. sclerophyllum*.

***Scleronema*** Benth. J. Proc. Linn. Soc., Bot. 6: 109. 1862. *Scleronema spruceanum* Benth., non *Scleronema* Brongn. & Gris Ann. Sci. Nat., Bot. 5 (2): 167. 1864, nom. illegit.

Trees (15–)20–40 m tall, unarmed, without buttresses. Trunks straight, bark brownish to reddish, striate with inconspicuous greenish striations, with evident lenticels; crown broad, branches verticillate, plagiotropic, rarely glaucous, with indumentum of fasciculate or stellate-rotate trichomes. Stipules deciduous, triangular, apices acuminate, acute or attenuate, indumentum with fasciculate or stellate-rotate trichomes. Seedling and adult trees leaves unifoliolate, petiolate, pulvinate, inarticulate; leaflets elliptic to oblong, elliptic to obovate, obovate, or oblong, chartaceous, coriaceous, rarely membranaceous, the tertiary veins mixed percurrent. Inflorescences fasciculate, axillary; bracteoles 3, triangular, deciduous. Flowers pedicellate; calyces (3–)5-lobed apically, gamosepalous; corolla dialypetalous, petals 5, reddish or white to slightly pinkish; staminal filaments fused into a cylindrical column longer than broad, glabrous, the free filaments (12–)16–30, relatively thick, glabrous; anthers monothecous, reniform; ovaries globose to oblate or ovoid, (2–)3-locular with 2 ovules per locule, superior, perigynous, densely covered with brown, fasciculate trichomes, styles glabrous, stigmas (2–)3-fidous, glabrous. Fruits indehiscent woody berries, globose to oblate, densely covered with brown, fasciculate trichomes, 1(–2)-seeded, the seeds globose to oblate and immersed in scarce, spongy endocarp.

A widely distributed genus in Amazonian rainforests, found in Brazil, Colombia, Peru, and Venezuela. Interestingly, we have seen no collections from the Guayanas. Its component species grow on well-drained, sandy or clayey soils not subject to seasonal flooding, at elevations of 50–600 m.s.m. (and rarely at elevations above 1000 m.s.m., e.g., *S. grandiflorum*).

***Scleronema grandiflorum*** Huber, Bol. Mus. Goeldi Paraense Hist. Nat. Ethnogr. 7: 299. 1913. *Catostemma grandiflorum* (Huber) Ducke, Arch. Jard. Bot. Rio de Janeiro 5: 165. 1930. Type: COLOMBIA. Cerro de Cupati. rio Caquetá (=Yapurá), 24 Nov 1912, *A. Ducke s.n.* (lectotype, designated by Ferreira et al. [17]: RB!; isolectotype: US [photo]!),

*Scleronema neblinense* Steyerm., Ann. Missouri Bot. Gard. 74: 645. 1987. Type: BRAZIL. Amazonas. Serra da Neblina, vicinity of Base Camp, Cano Tucano, Rio Cauaburí, 15 Nov 1965, *B. Maguire et al. 60181* (holotype: INPA!; MO [photo]!; isotypes: K [photo]!; NY [photo]!; RB!) **Syn. nov.**

*Scleronema guianense* Sandwith, Kew Bull. 1948: 304. 1948. Type: GUYANA. Bartica-Potaro road, 107 miles, in Clump Wallaba (Dicymbe) forest by creek, 15 Nov 1943, *D.B. Fanshawe 4227* (holotype: K [2 sheets] [photo]!; isotype: NY [photo]!) **Syn. nov.**

*Scleronema micranthum* (Ducke) Ducke, Trop. Woods 50: 37. 1937. ≡ *Catostemma micranthum* Ducke, Arch. Jard. Bot. Rio de Janeiro 5: 164, fig. 54. 1930. Type: BRAZIL. Amazonas. Manaus [Manáos], 13 June 1927, fl., *A. Ducke s.n.* (lectotype, designated by Ferreira et al. [17]: RB!; isolectotypes: INPA!; K [photo]!; MG [photo!]; MO [photo]!; NY [photo]!; NY [photo]!; P [photo]!; RB [only branches, leaves and flowers]!; S *p.p.* [only flowering branches] [photo]!; U [photo]!) **Syn. nov.**

Figure 9A–C,F–I

Trees 15–40 m tall. Branches not glaucous, densely covered with brown, fasciculate trichomes. Stipules 0.2–0.4 × 0.1–0.2 cm, apices acuminate, both surfaces not glaucous, and densely covered with brown, fasciculate trichomes. Seedling leaves with petioles (2.4–)4.9–9.6 cm long, sparsely covered with brown, fasciculate trichomes, leaflets 12.1–25.2(–31.5) × 5.7–11.3(–14.1) cm, membranaceous, elliptic to obovate, margins entire, bases rounded, apices caudate and mucronate, the adaxial surface glabrous, the abaxial surface with veins glabrous or sparsely covered with brown, fasciculate trichomes and without conspicuous stomata. Leaves of adult trees with petioles (1.4–)2.1–5.1(–6) cm long, not glaucous, densely or sparsely covered with brown, fasciculate trichomes, leaflets (6.8–)7.7–16.6(–24.2) × (3.5–)4.6–9.9(–12.1) cm, chartaceous to subcoriaceous, elliptic to oblong or obovate, margins entire, bases rounded, rarely cuneate or subcordate, apices rounded to retuse or obtuse, rarely acuminate and mucronate, veins of the adaxial surface glabrous or sparsely covered with hyaline, fasciculate trichomes, veins of abaxial surface sparsely covered with hyaline, fasciculate trichomes, without conspicuous stomata. Inflorescences 3–6-fasciculate, axillary; bracteoles 0.3–0.7 × 0.1–0.5 cm, apices acuminate, the adaxial surface sparsely covered with brown, fasciculate trichomes, the abaxial surface densely covered with brown, fasciculate trichomes. Floral pedicels (0.7–)1.1–2.6 cm long, densely covered with brown, fasciculate trichomes, hypanthia 0.05–0.1 × 0.3–0.4 cm, their adaxial surface glabrous; calyx tubes 0.3–0.4 × ca. 0.5 cm, apices 3–5-lobate, lobes ca. 0.6 × 0.3–0.5 cm and acute at apex, the adaxial surface of the calyx tube and lobes densely covered with hyaline, simple trichomes, the abaxial surfaces of the hypanthium, calyx tube, and calyx lobes with same indument as the pedicel; petals 1–1.5(–2) × 0.2–0.4 cm, obovate, apices rounded, white to slightly pinkish, both surfaces sparsely covered with brown, fasciculate trichomes, sometimes with a glabrous adaxial surface; staminal columns (0.1–)0.3–0.6 × (0.1–)0.2–0.3 cm, free filaments (16–)20–28, 0.6–0.9 cm long; ovaries ca. 0.1 × 0.2 cm, globose to oblate, 3-locular; styles 0.9–1.9 cm long; stigmas trifid. Immature fruits ca. 10 × 12 cm, 1(–2)-seeded; immature seeds ca. 5 × 7 cm.

*Representative Specimens Examined:* BRAZIL. Amazonas. Borba. Rio Madeira, 7 Nov 1935, fl., *A. Ducke s.n.* (MO, RB); Careiro. 8 July 1972, fl., *M.F. Silva et al. 278* (INPA, RB); Manaus. 6 July 1963, fl., *W.A. Rodrigues & D.F. Coêlho 5264* (COL, INPA, RB); AM-010, 13 May 1972, fl., *A.A. Loureiro & O. Pires s.n.* (INPA, RB); 17 May 1988, fl., *D. Coêlho 48-D* (INPA, RB); BR-174, 22 Mar 1993, fl., *W.A. Rodrigues 11053a* (INPA, RB); 20 Sep 1976, fr., *C.D.A. da Mota 688* (COL, INPA, MO, RB); Distrito Agropecuário da SUFRAMA, June 1980, fl., *J.L. Santos & B. Zimmerman 516* (INPA); 1 June 1992, fl., *A.A. Oliveira & R.M. Cardoso 460* (INPA, RB); 1997, fr., *M.C. Lemos 11* (INPA, RB); Reserva Florestal Adolpho Ducke, 29 Oct 2021, seed., *C.D.M. Ferreira et al. 829* (RB); 29 Oct 2021, fr., *C.D.M. Ferreira et al.* 834 (RB); Presidente Figueiredo. 27 May 1974, fl., *W.A. Rodrigues et al. 9359* (INPA, RB); 10 May 1995, st., *C.C.N. Araújo s.n.* (INPA, RB); 6 Nov 2021, st., *C.D.M. Ferreira et al. 858* (RB); Reserva Biológica do Uatumã, 10 July 2007, fl., *S. Sakagawa & J.R. Mesquita 422* (INPA, RB); 18 Sep 2017, fl., *W. Castro et al. 2487* (INPA, RB); 8 July 2018, fl., *L.O. Demarchi 1240* (INPA, RB); 19 July 2015, fl., *F.M. Costa et al. 2090* (INPA, RB); 7 Dec 1982, fl., *F.R. Silva 70* (INPA, RB); 24 Oct 1983, fl., *J.L. Santos 572* (INPA, RB); 2 July 1986, fl., *W. Thomas et al. 5281* (US); Rio Preto da Eva., 10 July 1974, fr., *W.A. Rodrigues & A.A. Loureiro 9442* (INPA, MO, RB); Santo Antônio do Içá. 21 Nov 1986, fl. and fr., *C.A. Cid et al. 8489* (MO, NY, US); São Gabriel da Cachoeira. Rio Miuá, 3 Nov 2021, st., *C.D.M. Ferreira et al. 855* (RB); COLOMBIA. Amazonas. Araracuara. Sep 1992, st., *A. Posada et al. 1676* (HUA); 6 Nov 1990, seed., *E. Alvarez et al. 822* (COAH); 10 Mar 2001, seed., *A. Duque et al. 6121* (COL); 2 Mar 2001, seed., *A. Duque et al. 5659* (COL); 2 Apr 2001, seed., *A Duque et al. 6790* (COL); Chorrera, 10 Oct 2018, seed., *D. Cárdenas et al. 50509* (COAH); Resguardo indígena Muinane, June 1992, st., *A.J. Duque et al. 491* (HUA); Tarapacá. 6 Mar 1999, st., *R. López et al. 4852* (COAH); 1 July 1992, st., *A. Rudas et al. 4563* (FMB, MO); 7 Mar 1999, st., *R. López et al. 4996* (COAH); 9 Aug 1994, st., *D. Cárdenas & H. Martínez 5319* (COL); Caquetá. 13 Jan 1995, fl., *A. Dulmen AvD310* (COAH, MO); 12 Apr 1993, seed., *N. Hernández et al. CHI-800* (COL); 11 Sep 2001, st., *A. Duque et al. 9464* (COAH); 18 Aug 2001, st., *A. Duque et al. 8485* (COAH); Parque Nacional Natural Serranía de Chiribiquete, 15 Feb 2001, fl., *H. Mendonza et al. 10185* (FMB); 15 Apr 2001, st., *s/col. s.n.* (FMB); 15 Feb 2001, st., *H. Mendonza et al. 12261* (FMB); 20 Jan 2000, st., *H. Mendonza et al. 13941* (FMB); 27 May 2000, st., *J.F. Phillips 307* (COL); Estacíon Puerto Abeja. 15 Jan 2000, fl., *A.M. Eusse & J.A. Montes 921* (COAH); 13 Sep 1999, fl., *A.M. Eusse & J.A. Montes 527* (COAH); Leticia. Estacíon Biológica Zarife, 25 Dec 2005, st., *P. Trujillo et al. 2994* (JAUM); 9–12 Dec 2007, st., *A. Robles et al. 532* (FMB); Parque Nacional Natural Amacayacu, 28 Mar 1992, seed., *A. Rudas et al. 4006* (COL, HUA); 7 Aug 1994, st., *D. Cárdenas & H. Martínez 5160* (COL); Putumayo. 3 Sep 2016, st., *Y. Gutiérrez et al. 242* (COAH); Vaupés. 21 May 2009, st., *S. Castro et al. 1649* (COAH); 23 July 2006, st., *D. Cárdenas et al. 19053* (COAH); 23 Mar 2009, fr., *D. Cárdenas et al. 22144* (COAH); VENEZUELA. Amazonas. 29 Mar 2000, st., *P.E. Berry & G. Aymard 7346* (MO); Atabapo. Feb 1992, fr., *A. Chaviel 326* (MO); Cerro Neblina. 4 Mar 1984, fl. and fr., *A. Gentry & B. Stein 47148* (MO, NY). See additional specimens examined in Appendix A.

*Common names: dua-haarana* (Baniwa); *envirão, envirão-branco, cardeiro, cedro-bravo* (Portuguese); *castaño* (Spanish); *jagarai* (Witoto); *mamao-rana* (possibly Tupi); *mandí* (Andoque); *riiribagué* (Miraña); *kema, yolombo* (unknown).

**Figure 10:**
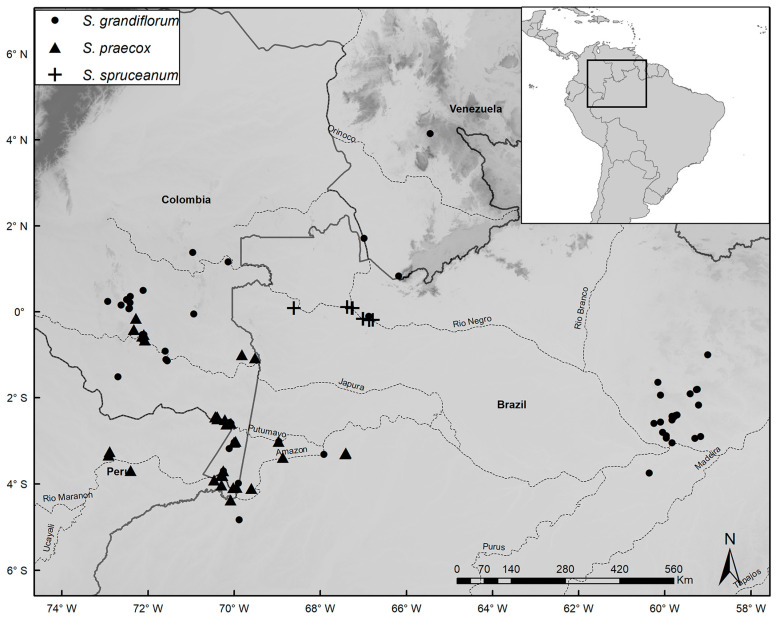
Geographic distribution of species of the genus *Scleronema*.

**Figure 1:**
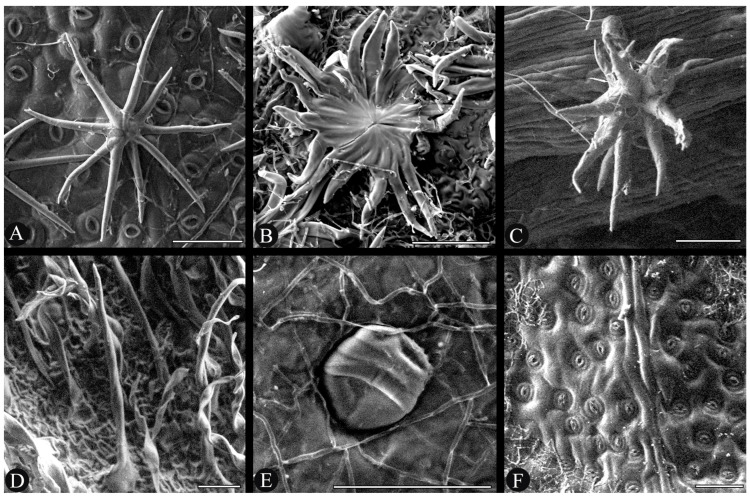
Types of trichomes observed on *Aguilaria*, *Catostemma*, and *Scleronema*. (**A**) Stellate-rotate trichome, *S. praecox*. (**B**) Dentate-lepidote trichome, *A. excelsa*. (**C**) Fasciculate trichome, *S. spruceanum*. (**D**) Simple trichome, *C. lanceolatum*. (**E**) Glandular trichome, *A. excelsa*. (**F**) Dense, conspicuous stomata, *S. spruceanum*. Scale: 50 µm.

**Figure 5:**
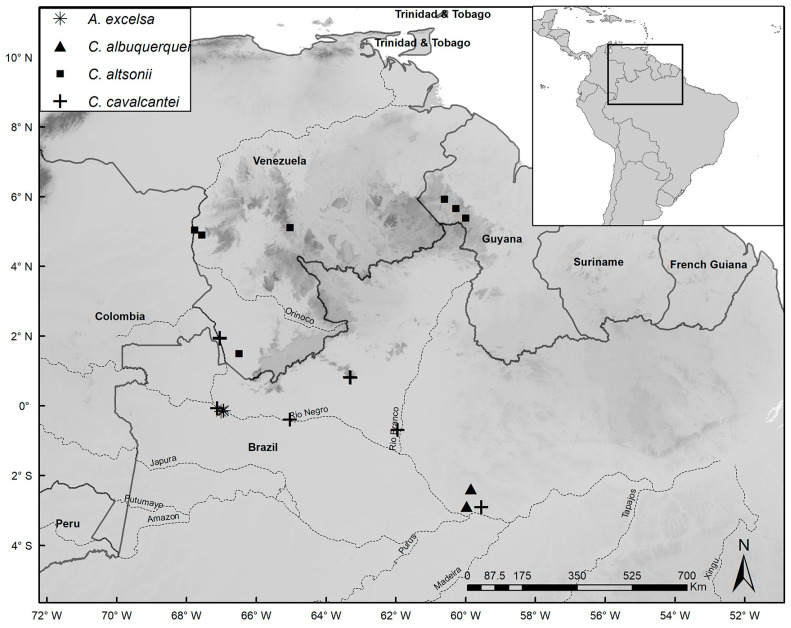
Geographic distribution of *Aguiaria excelsa*, *Catostemma albuquerquei*, *C. altsonii*, and *C. cavalcantei*.

**Figure 6:**
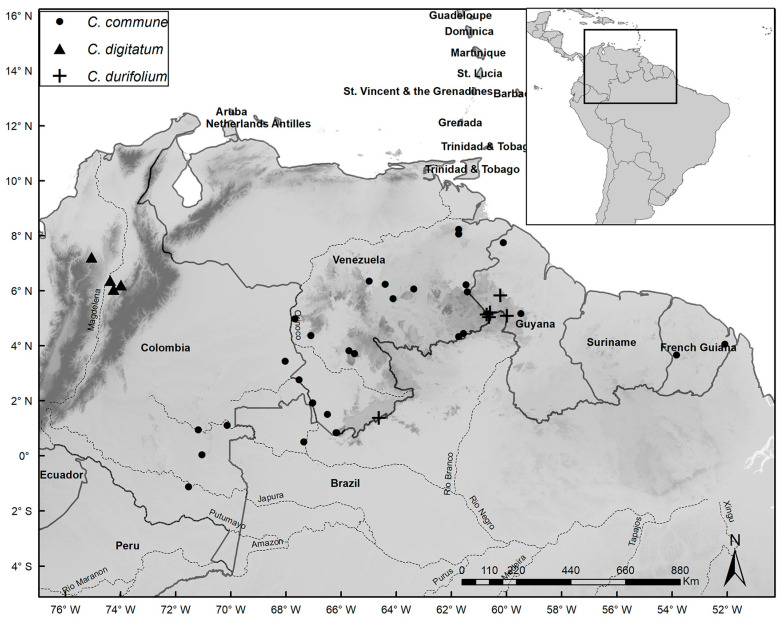
Geographic distribution of *Catostemma commune*, *C. digitatum*, and *C. durifolium*.

**Figure 7:**
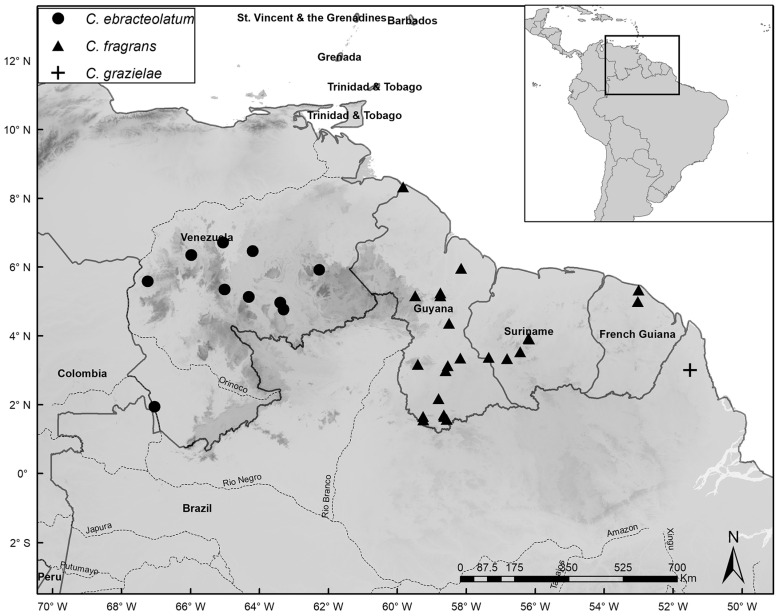
Geographic distribution of *Catostemma ebracteolatum*, *C. fragrans*, and *C. grazielae*.

**Figure 8:**
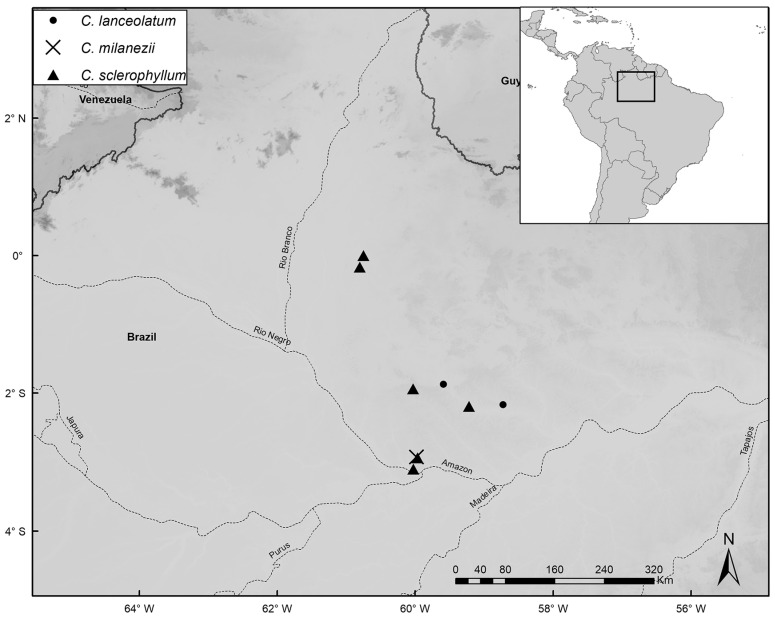
Geographic distribution of *Catostemma lanceolatum*, *C. milanezii*, and *C. sclerophyllum*.

*Distribution and Habitat:* This species occurs widely in Brazil, Colombia, and Venezuela on well-drained clayey (or rarely sandy) soils in areas not subject to flooding, at elevations of 50–600 m.s.m. (and rarely at elevations of ca. 1600 m.s.m: a specimen from Putumayo, Colombia; *Y. Gutiérrez et al. 242*) (Figure 10).

*Phenology:* Flowering specimens were collected in all months of the year and fruiting specimens from March to November.

*Conservation Status:* This is a widely distributed species with an EOO greater than 20,000 km^2^ and several known localities of occurrence, including protected areas (Estacíon Biológica Zarife, Parque Nacional Natural Amacayacu, Parque Nacional Natural Serranía de Chiribiquete, Reserva Biológica do Uatumã, Reserva Florestal Adolpho Ducke, and Resguardo indígena Muinane). Therefore, *S. grandiflorum* is herein considered LC.

*Notes:* This differs from the other species of the genus by the branches and petioles covered by fasciculate trichomes (vs. stellate-rotate trichomes), and white to slightly pinkish petals (vs. reddish).

*Catostemma micranthum* was published by Ducke in 1930 [19] and later transferred by him to *Scleronema* [20]. *Scleronema guianensis* was published by Sandwith in 1948 [4]. Both taxa are here proposed as new synonyms of *S. grandiflorum*, described by Huber in 1913 [21]. Based on type specimens, we concluded that there are no significant differences to support them as distinct species. As for *S. neblinense*, the name was described by Steyermark in 1987 [7] and later treated as a synonym by Alverson & Steyermark [18] and Ferreira et al. [17], consistent with the treatment here.

***Scleronema praecox*** (Ducke) Ducke, Trop. Woods 50: 37. 1937. *Catostemma praecox* Ducke Arch. Jard. Bot. Rio de Janeiro 5: 163. 1930. Type: BRAZIL. Amazonas. São Paulo de Olivença, 18 Oct 1927, *A. Ducke s.n.* (lectotype, designated by Ferreira et al. [17]: RB!; isolectotypes: K [photo]!; NY [photo]!; P [photo]!; P [photo]!; RB!; RB [fragment]!; S [photo]!; U [photo]!; U [photo]!; [photo]!). COLOMBIA. Vaupés. Taraira. Estacíon Biológica Caparú, a 3 km al norte del Lago Taraira. Terraza, 200 m.s.m., 14 Apr 1988, *S. Defler 5248* (epitype, designated by Ferreira et al. [17]: COAH [photo]!).

Figure 9D

Trees 30–40 m tall. Branches not glaucous, densely covered with brown, stellate-rotate trichomes. Stipules ca. 0.8 × 0.3 cm, apices acute, both surfaces not glaucous, and densely covered with brown, stellate-rotate trichomes. Seedling leaves with petioles 4.9–6 cm long, sparsely or densely covered with hyaline, simple, stellate-rotate and glandular trichomes, leaflets 16.5–26.3 × 6–8.2 cm, membranaceous, elliptic, margins entire, bases rounded, apices caudate and mucronate, the adaxial surface sparsely with brown glandular trichomes and veins sparsely covered with hyaline simple, stellate-rotate, and brown glandular trichomes, the abaxial surface densely covered with brown glandular trichomes and veins sparsely covered with brown and hyaline stellate-rotate and glandular trichomes, bearing dense, conspicuous stomata. Leaves of adult trees with petioles (1.3–)1.9–3.5(–4.8) cm long, not glaucous, densely covered with brown, stellate-rotate trichomes, leaflets (12.5–)15.4–23.1 × (6.6–)8.8–10.6 cm, chartaceous, elliptic to obovate or oblong, margins entire, bases rounded or subcordate, apices acuminate or rounded and mucronate, veins of adaxial surface densely covered with brown, stellate-rotate trichomes, the abaxial surface densely covered with brown, stellate-rotate trichomes, with dense and conspicuous stomata. Inflorescences 2–3-fasciculate, axillary; bracteoles ca. 0.7 × 0.2 cm, apices acute, both surfaces densely covered with brown, fasciculate trichomes. Floral pedicels 1.9–2.3 cm long, densely covered with brown, fasciculate trichomes; hypanthia 0.2–0.3 × 0.3–0.4 cm, their adaxial surface glabrous; calyx tubes 0.3–0.4 × 0.6–0.7 cm, apices 5-lobate, lobes 0.2–0.4 × 0.2–0.3 cm, lobe apices acute, the adaxial surfaces of the calyx tube and lobes densely covered with brown, simple trichomes, the abaxial surfaces of the hypanthium, calyx tube, and calyx lobes with same indument as the pedicel; petals 0.9–1.3 × ca. 0.4 cm, obovate, apices rounded or obtuse, reddish, and both surfaces densely covered with hyaline, fasciculate trichomes; staminal columns 0.1–0.3 × 0.1–0.2 cm, free filaments 20–30, 0.5–0.8 cm long; ovaries ca. 0.3 × 0.2 cm, globose to oblate, 3-locular; styles ca. 1.3 cm long; stigmas trifid. Immature fruits ca. 7 × 9 cm, 1-seeded; immature seeds ca. 4 × 5 cm.

*Representative Specimens Examined:* BRAZIL. Amazonas. Esperança. 7 Oct 1942, fl. and fr., *A. Ducke 1085* (IAN, INPA, MG, MO, RB, US); Benjamim Constant. 15 Oct 1956, fl., *E.M. Drees 2* (IAN, INPA, MG); 16 Oct 1956, st., *E.M. Drees 13* (INPA, MG, RB); 19 Oct 1956, st., *E.M. Drees 16* (MG, INPA); Jutaí. Reserva Extrativista do Rio Jutaí, 10 Sep 2005, fl., *M.A.D. Souza et al. 1933* (INPA, RB); São Paulo de Olivença. 24 Nov 1986, fr., *C.A. Cid et al. 8576* (INPA, MO, US); 26 Octr–11 Dec 1986, fl., *B.A. Krukoff 8844* (MO, US); 17 Oct 1942, fl., *A. Ducke 1086* (IAN, MG, MO, US); COLOMBIA. Amazonas. 10 Sep 1988, *M. Sanchez et al. 1099* (COAH, COL); Parque Nacional Natural Amacayacu, 20 Jan 1988, fr., *A. Gentry & J.A. Villa-Lopera 60790* (COL); 22 Feb 1991, fr., *J. Pipoly et al. 13340* (MO); 16 Aug 1996, st., *A. Posada et al. 1977* (COAH); Araracuara. 19 Oct 1990, st., *D. Restrepo et al. 38* (COAH); 8 Oct 1992, st., *A. Duque & T. Matapi 804* (HUA); 6 Mar 1993, st., *A. Duque et al. 1012* (HUA); 24 Jan 1989, st., *A. Gentry & M. Sanchez 65014* (MO); 27 Nov 1997, st., *A.C. Londoño & E. Alcaraz 2424* (JAUM); 4 Nov 1989, st., *C. Londoño et al. 1148* (NY); Caquetá. 15 Nov 1995, st., *N. Rodríguez & D. Cárdenas 39* (COAH); La Pedrera. Resguardo Indígena Yaihojé-Apaporis, 4 Dec 2018, st., *A. Álvarez et al. 2851* (COAH); Leticia, 9 Sep 1963, fl., *D.D. Soejarto & H. Cardozo 663* (COL); Parque Nacional Natural Amacayacu, s.d., st., *J.S.B. Silva et al. 2194* (COAH); 19 Mar 1992, st., *A. Rudas et al. 3236* (COL, FMB, HUA, MO); 20 Mar 1992, st., *A. Rudas et al. 3255* (COL, FMB, HUA, MO); 25 Mar 1992, *A. Rudas et al. 3704* (COL, FMB, HUA); Resguardo Indígena de Nazareth, 24 Apr 2008, st., *C. Lopera et al. 76* (COL); 23 Apr 2008, st., *C. Lopera et al. 75* (COL); Resguardo indígena Ticuna-Uitoto, 9 Apr 2003, st., *J.C. Arias-G. 1197* (COAH); San Pedro de Tipisca. 12 Oct 1996, st., *A. Posada et al. 2994* (COAH); Tarapaca. 17 Mar 1999, st., *R. Lopéz et al. 5568* (COAH); 20 May 2008, fr., *J.C.J. Luna & V.H.M. Parra 1* (MEDEL); 15 May 2008, fr., *J.C.J. Luna & V.H.M. Parra 3* (JAUM); 20 May 2008, fr., *J.C.J. Luna & V.H.M. Parra 3A* (JAUM); 9 Aug 1994 st., *D. Cárdenas & H. Martínez 5160* (COL); 6 Mar 1999, st., *R. López et al. 4881* (COAH); 15 Dec 1998, st., *R. López et al. 4421* (COAH); Parque Nacional Natural Amacayacu, 13 July 1992, st., *A. Rudas et al. 5240* (COL, FMB, MO); 6 July 1992, st., *A. Rudas et al. 4853* (COL, FMB, MO); Santa Lucía. 9 Aug 1994, st., *D. Cárdenas & H. Martínez 5305* (COL); Vaupes. Estácion Biológica Caparú, Mar 1990, st., *S. Defler 55* (COAH, MO); 25 Mar 1988, fl., *S. Defler 49* (COAH, MO, FMB); Estacíon Biológica Mosiro Itajura, 10 May 2004, fr., *L. Clavijo-R & W. Tanimuka 905* (COAH, COL); Jotabeyá. 28 Mar 2009, st., *D. Cárdenas et al. 22252* (COAH); PERU. Loreto. 1980, st., *S. Barrier s.n.* (MO); 1 Feb 1995, fl., *C. Grández & J. Devlin 5958* (MO); Paucarillo Reserve, 3 Nov 2001, fl., *J. Choo 46* (MO); Sucusari. 24 Feb 1991, st., *J. Pipoly et al. 13669* (COL, MO); 5 July 1983, fl., *A. Gentry et al. 42623* (MO); 12–24 Feb 2001, st., *A. Monteagudo et al. 2319* (CUZ). See additional specimens examined in Appendix A.

*Common names: castanha-branca*, *castanha-de-paca*, *castanha-de-paca-vermelha*, *envira-de-veado* (Portuguese); *castaño*, *castaño-rojo*, *niguno* (Spanish); *júbuó*, *maario* (Mui).

*Distribution and Habitat: Scleronema praecox* occurs in Brazil, Colombia, and Peru on well-drained clay, or rarely sandy, soils in areas not subject to flooding, at elevations of 90–300 m.s.m. (Figure 10).

*Phenology:* Flowering specimens were collected from July to October and fruiting specimens from October to May.

*Conservation Status:* A widely distributed species with an EOO greater than 20,000 km^2^, several known occurrence localities, and specimens registered in protected regions (Estácion Biológica Caparú, Estacíon Biológica Mosiro Itajura, Parque Nacional Natural Amacayacu, Paucarillo Reserve, Reserva Extrativista do Rio Jutaí, Resguardo Indígena de Nazareth, Resguardo indígena Ticuna-Uitoto, and Resguardo Indígena Yaihojé-Apaporis), *S. praecox* is herein considered LC.

*Notes:* This differs from the other species of the genus by its leaflets with brownish adaxial surfaces with strongly evident veins. Its petals are reddish, as in *S. spruceanum*, which differs from *S. grandiflorum* with white to slightly pinkish petals.

***Scleronema spruceanum*** Benth., J. Proc. Linn. Soc. Bot. 6: 109. 1862. *Catostemma spruceanum* (Benth.) Bakh. Bull. Jard. Bot. Buitenzorg (3)6: 218. 1924. Type: BRAZIL. North Brazil, on the Rio Uaupès, in Caatingas about the cataracts of Jauaratè, Oct 1852–Jan 1853, *R. Spruce 2548* (lectotype, designated by Ferreira *et al.* [17]: K [photo]!; isolectotypes: C [photo]!; F [fragment] [photo]!; F [fragment] [photo]!; MPU [photo]!; P [photo]!).

Figure 9E

Trees 20–30 m tall. Branches usually glaucous, densely covered with brown, stellate-rotate trichomes. Stipules ca. 0.2 × 0.1 cm, apices attenuate, both surfaces not glaucous, and densely covered with brown, stellate-rotate trichomes. Seedling leaves with petioles 4.1–6.9 cm long, sparsely covered with brown, glandular trichomes, leaflets 12.4–18.4 × 4.4–7.1 cm, chartaceous, elliptic to narrow elliptic, margins entire, bases rounded, apices caudate and mucronate, the adaxial surface glabrous, the abaxial surface sparsely covered with brown, glandular trichomes and bearing dense, conspicuous stomata. Leaves of adult trees with petioles (1.7–)2.2–4.2 cm long, usually glaucous, sparsely covered with brown, stellate-rotate trichomes, leaflets (10.2–)13.3–15.4 × 5.8–7.8 cm, coriaceous, elliptic to obovate, margins entire, bases rounded or subcordate, apices acuminate or attenuate and mucronate, the adaxial surface glabrous, the abaxial surface glabrous with veins sparsely covered with hyaline, fasciculate trichomes and dense, conspicuous stomata. Inflorescences 5-fasciculate, axillary; bracteoles ca. 0.1 × 0.1 cm, apices acute, both surfaces densely covered with brown, fasciculate trichomes. Floral pedicels (0.7–)1–2.3 cm long, densely covered with brown, fasciculate trichomes; hypanthia ca. 0.1 × 0.3–0.4 cm, their adaxial surface glabrous; calyx tubes ca. 0.3 × 0.4–0.5 cm, apices 5-lobate, lobes 0.4–0.5 × ca. 0.3 cm, lobe apices acute, the adaxial surface of the calyx tube and lobes densely covered with hyaline, simple trichomes, the abaxial surfaces of the hypanthium, calyx tube, and calyx lobes with same indument as the pedicels; petals 1.2–1.4 × ca. 0.3 cm, obovate, apices rounded, reddish, glabrous on adaxial surface, densely covered with hyaline, fasciculate trichomes on abaxial surface; staminal columns 0.2–0.4 × ca. 0.1 cm, free filaments 12–26, 0.6–0.9 cm long; ovaries ca. 0.2 × 0.2 cm, ovoid, (2–)3-locular; styles 1.2–1.5 cm long; stigmas (2–)3-fid. Immature fruits ca. 6 × 5 cm, 1-seeded; immature seeds ca. 3 × 2 cm.

*Specimens Examined:* BRAZIL. Amazonas. São Gabriel da Cachoeira. 8 Mar 1975, fr., *J.M. Pires & L.R. Marinho 15753* (IAN); 2011, seed., *M.M. Pombo et al. 274* (EAFM); 9 Sep 2010, seed., *M.R.M. Amaral et al. 160* (EAFM); Feb 1959, seed., *W. Rodrigues 1016* (INPA); Carapanã. 16 Oct 1987, fl., *C. Farney et al. 1750* (INPA, MG, MO, RB, US); 19 Oct 1987, fl., *D.C. Daly et al. 5454* (INPA, MG, NY, US); Curicuriary. 23 Nov 1929, fl., *A. Ducke s.n.* (US, RB); 22 Feb 1936, fr., *A. Ducke s.n.* (RB); estrada perimetral Norte. 6 Apr 2013, seed., *D. Cardoso et al. 3405* (INPA); Rio Içana; Comunidade de Jandu Cachoeira. July 2007, *J. Stropp & P. Assução 281* (EAFM); Taraquá. 5 Mar 1959, fr., *J.S. Rodrigues 202* (IAN); Itacoatiara Mirim. 31 Oct 2021, fl., *C.D.M. Ferreira et al. 837* (RB); 31 Oct 2021, seed., *C.D.M. Ferreira et al. 838* (RB); 31 Oct 2021, fl., *C.D.M. Ferreira et al. 841* (RB); 29 Mar 2013, st., *D. Cardoso et al. 3319* (INPA); July 2007, st., *J. Stropp & P. Assução 284* (EAFM); Waupes. 29 Sep 1935, fl., *A. Ducke s.n.* (US, RB); 9 Apr 1952, fl., *R.L. Fróes 28244* (IAN, MG, UB); 2 Mar 1975, fr., *M.R. Cordeiro 421* (IAN).

*Common names: dua* (Baniwa); *envirão, envirão-vermelho* (Portuguese).

*Distribution and Habitat:* A species restricted to the municipality of São Gabriel da Cachoeira in the upper Rio Negro, Amazonas, Brazil. It grows on well-drained sandy soils not subject to seasonal flooding, at elevations of 60–80 m.s.m. (Figure 10).

*Phenology:* Flowering specimens were collected from September to April and fruiting specimens from February to March.

*Conservation Status:* Although it is a species of restricted distribution, it exceeds 20,000 km^2^ of EOO and is known from more than ten localities, so *S. spruceanum* is herein considered LC.

*Notes:* This species differs from the others of the genus by its glaucous branches and petioles, and its coriaceous leaflets. Its petals are reddish, as in *S. praecox*, which differs from *S. grandiflorum* with white to slightly pinkish petals.

Uncertain species

*Catostemma lemense* Sanoja, Acta Bot. Venez. 27: 85. 2004. Type: VENEZUELA. Bolívar. Gran Sabana. Bosque 30–35 m de alto a orilla de la carretera El Dorado-Santa Elena de Uairén, km 128, frente a la cantera Dell’Acqua, 1435 m.s.m, 26 Oct 1997, *E. Sanoja et al. 4750* (holotype: GUYN, not seen).

*Catostemma lemense* was published by Sanoja in 2004 [22] based on specimens collected in Sierra de Lema and Ptari-te, Gran Sabana, Bolívar, Venezuela. The author cited the greater rigidity and larger size of the seedling leaves to differentiate the species from *C. commune*, and indicated that the fruits are two- to four-times larger. Flowers were not described and all type specimens are sterile except for the holotype, which is in fruit. We managed to analyze only a few paratypes (*Steyermark 59827*, *60043*; *Hernández et al. 983; Sanoja et al. 4958*, *4970*, *6377*, *6606*), and as indicated by Sanoja, they all show great similarities to *C. commune*. The few differences pointed out by the author could easily be explained by the high morphological plasticity of *C. commune*, a species with a wide distribution. We reported this view in a previous publication [17], where we presented the nomenclatural revision of the group. Dr. Sanoja has collected flowering specimens of this species (pers. comm.), but due to political issues in Venezuela, herbaria where these specimens were deposited are deactivated or inaccessible. Thus, we could only analyze a photograph of the living flowers provided by Dr. Sanoja. While this was of significant value for analysis, it did not allow us to obtain detailed information on the androecium and ovary or to measure and describe the indumentum. Therefore, we maintain *C. lemense* as an uncertain species, hoping that future fieldwork and analyses can elucidate its status.

## Data Availability

The original contributions presented in this study are included in the article/Appendix A. Further inquiries can be directed to the corresponding author.

## Appendix A

### Additional Specimen List

***C. commune***: seed. *A. Gentry & B. Stein 46758* (MO); st. (*p.p. S. grandiflorum* fruit) *47038*, seed. *46807* (MO, NY); fl., *A.L. Bernardi 898*, fl., *2097*, st, *2897* (NY); st. *A. Roa 379* (COAH); fr. *B. Trujíllo & D. Martínez 21329* (MO); st., *E. Acero & R. Rodriguez 964* (COAH); seed., *Hernandez 1731* (VEN); fr. and seed. *H. Leng 416* (NY); fr., *J.A. Steyermark 74781*, fr., *86640* (VEN); fr., *86715*(NY); fr., *87583*, st., *112934* (MO); st., *L. Bernardi & J.J. Buza 7529* (NY); st. *L.E. Urrego* et al. *643*, st. *788* (COAH); st. *M. Sanchez* et al. *379* (COAH); st. *P.A. Palacios* et al. *1894* (COL); st. *R. Liesner 25054* (MO); st. *S. Bergeron 254-79* (COAH); st. *S. Bergeron & L. Román 462* (COAH); seed. *T.V. Andel 303* (COAH, COL); seed. *505* (COAH). ***C. fragrans***: fl., *A.C. Persaud 134* (NY); fr., *K.M. Redden* et al. *5277* (NY); fr., *M.F. Prèvost 2160* (NY); fr., *M.J. Jansen-Jacobs* et al. *1929* (MG, MO, NY); fr., *1978* (MO); fr., *R. Evans* et al. *1940* (IAN, NY); fr., *2003* (IAN, RB); seed. *S. Mori* et al. *8115* (NY). ***S. grandiflorum***: fl., *A.A. Oliveira & R.M. Cardoso 460* (US); st., *A.B. Scabin* et al. *140* (EAFM); fr., *A.C.A. Oliveira* et al. *300* (INPA, RB); fl., *A. Ducke 1269* (MG, US); fr., *A. Gentry & B. Nelson 69236* (MO); st., *A.J. Duque* et al. *172*, seed., *564* (COL); fr., *A. Knob* et al. *729* (HUAM); fl., *1071* (HUAM); fr., *A.P. Silva s.n.* (US); fl., *A.V. Dulmen 178* (COAH, COL); fl., *A. Webber* et al. *1059* (HUAM); fl., *C.A. Cid 7001* (INPA, MO, RB); fr., *C.A. Cid* et al. *996* (RB, US); fl. and fr., *C.C. Berg* et al. *P18153* (MG, US); fr., *C.D.M. Ferreira & G.P. Viana 870* (RB); fl. and fr., *C. Dick 183* (INPA, NY, MO, US); fl., *Corner 103* (COL, IAN); seed., *C. Rotta 59* (COL); seed., *C. Rotta & P. Miraña 357* (COAH, COL); st., *D. Cárdenas & H. Martínez 5319* (COL); fl., *D. Coêlho s.n.* (INPA, RB); fl., *3900* (MG); st., *D.M. Marra* et al. *113* (EAFM); seed., *1211* (EAFM); st., *1725* (EAFM); fl., *E. Ferreira 58-292* (IAN, INPA); fl., *E. Palheta s.n.* (INPA, US); fr., *E.C. Pereira s.n.* (INPA, NY); fl., *E.S.C. Gurdel & L.C.B. Lobato 783* (IAN, MG); fr., *F. Bisby* et al. *P18121* (INPA, MG); fr., *F. Mello & J. Ribamar 68* (COL); fl., *G.T. Prance* et al. *2182* (MG, MO); fr., *3822* (INPA, MO, US); fr., *9037* (INPA, MG, US); fl., *3041*(INPA, MG, US); fl., *3083* (INPA, MO, MG, US); fl., *G.T. Prance & F. Ehrendorfer, 22739* (COL, INPA, NY, US); fl., *I.M. Idrobo* et al. *11304* (HUA, NY); fl., *J. Aluisio 49* (INPA, US); fl., *J.E.L.S. Ribeiro* et al. *1013* (INPA, US); seed., *J.F. Phillips 69*, st., *391* (COL); fl. and fr., *J.G. Carvalho-Sobrinho & J.R. Mesquita 1584* (INPA, RB); fr., *J.L. Santos & B. Zimmerman 501* (INPA, RB); fl., *J.R. Mattos* et al. *514* (RB, US); fl., *L. Eggers* et al. *s.n.* (INPA, US); st., *L.E. Urrego* et al. *1039* (NY); st., *L. Mars* et al. *s.n.* (INPA, US); seed., *L.O. Demarchi & L.C. Moura 211* (EAFM); fl., *M.A. Freitas* et al. *100*, fl., *430* (US); fl. and fr., *M.J.R. Pereira* et al. *s.n.* (MO, US); fr. *M. Pacheco* et al. *39* (US); st., *M. Sánchez* et al. *766*, st., *1057* (COAH); fr., *N.A. Rosa 533* (IAN); st., *N. Hernández* et al. *CHI-1500* (COL); fl., *O. Cunha & Raimundo 307* (HUAM); st., *P.A. Palacios* et al. *1808* (COAH); fr., *R. Bilby* et al. *593* (HUAM); st., *R. Liesner 16539* (MO, VEN); st., *R. López* et al. *4996* (COAH); seed., *Rudas* et al. *4215* (COL, HUA, COL); fl., *S. Mori & M. Cardoso 20712* (INPA, MO, US, VEN); st., *S.C. Amoêdo* et al. *91* (EAFM); st., *T.V. Andel* et al. *28* (COAH); fl., *W.A. Rodrigues 531* (MG); fl., *5364* (INPA, RB); fl., *5457* (COL, INPA); fl., *W.A. Rodrigues & O.P. Monteiro 6761* (INPA, RB); fl., *8194* (COL, INPA); fl., *W.A. Rodrigues & Osmarino 5979* (INPA, RB); fr. *W.W. Thomas 3348* (MO, NY). ***S. praecox***: seed., *A. Roa 672* (INPA); st., *A. Rudas* et al. *4332* (COL, FMB, MO); st., *4364*, st., *4450*, st., *5281*, st., *5572* (COL, FMB); st., *A. Monteagudo* et al. *1906*, st., *1960*, st., *1999*, st., *2006*, st., *2181* (CUZ); st., *2208* (AMAZ, CUZ); fl., C. Londoño et al. 1191 (COAH, NY); st., *J. Pipoly* et al. *13218*, st., *14630* (COL); st., *13340*, st., *13512*, st., *14369* (COL, MO); st., *R. Vásquez & N. Jaramillo 15997* (MO); st., *S. Defler 41*, st., *42*, st., *44*, st., *45*, st., *47* st., *56*, (MO); st., *43*, st., *51* (COAH, MO); fl., *46* (FMB, MO); fl., *48*, fl., *50* (COAH, FMB, MO).

## References

[1] Carvalho-Sobrinho J.G., Alverson W.S., Alcantara S., Queiroz L.P., Mota A.C., Baum D.A. (2016). Revisiting the phylogeny of Bombacoideae (Malvaceae): Novel relationships, morphologically cohesive clades, and a new tribal classification based on multilocus phylogenetic analyses. Mol. Phylogenetics Evol..

[2] Sandwith N.Y. (1928). New species from British Guiana. Bull. Misc. Inf. (Royal Gard. Kew).

[3] Sandwith N.Y. (1931). Contributions to the flora of tropical America-IV, The Baromallis of British Guiana. Kew Bull..

[4] Sandwith N.Y. (1948). Contributions to the Flora of Tropical America: XLVIII. Notable Additions to the Flora of British Guiana. Kew Bull..

[5] Ducke A. (1935). *Aguiaria*, novo gênero de Bombacáceas, a árvore maior do Alto Rio Negro. An. Acad. Bras. De Ciências.

[6] Paula J.E.P. (1969). Estudos sobre Bombacaceae—I. Contribuição para o conhecimento dos gêneros *Catostemma Benth*. e *Scleronema Benth*. da Amazônia Brasileira. Ciência Cult..

[7] Steyermark J.A. (1987). Flora of the Venezuelan Guayana III. Ann. Mo. Bot. Gard..

[8] Thiers B. Index Herbariorum: A Global Directory of Public Herbaria and Associated Staff. New York Botanical Garden’s Virtual Herbarium. http://sweetgum.nybg.org/ih/.

[9] Beentje H. (2016). The Kew Plant Glossary: An Illustrated Dictionary of Plant Terms.

[10] Payne W.W. (1978). A glossary of plant hair terminology. Brittonia.

[11] Theobald W.L., Krahulik J.L., Rollins R.C., Metcalfe C.R., Chalk L. (1979). Trichome description and classification. Anatomy of the Dicotyledons. I..

[12] Ellis B., Daly D., Hickey L.J., Johnson K., Mitchell J.D., Wilf P., Wing S.L. (2009). Manual of Leaf Architecture—Morphological Description and Categorization of Dicotyledonous and Net-Veined Monocotyledonous Angiosperms.

[13] Ferreira C.D.M., Alverson W.S., Demarchi L.O., Bovini M.G., Baumgratz J.F.A. (2023). *Catostemma lanceolatum* (Malvaceae/Bombacoideae/Adansonieae), a new species from the Brazilian Amazon. Phytotaxa.

[14] ESRI (2011). ArcGIS Desktop: Release 10.

[15] IUCN Guidelines for Using the IUCN Red List Categories and Criteria. Version 16. Cambrigde UK. https://www.iucnredlist.org/documents/RedListGuidelines.pdf.

[16] Cardoso D., Carvalho-Sobrinho J.G., Zartman C.E., Komura D.L., Queiroz L.P. (2015). Unexplored Amazonian diversity: Rare and phylogenetically enigmatic tree species are newly collected. Neodiversity.

[17] Ferreira C.D.M., Alverson W.S., Bovini M.G., Baumgratz J.F.A. (2023). The nomenclature of the Catostemma clade (Malvaceae/Bombacoideae/Adansonieae). Brittonia.

[18] Alverson W.S., Steyermark J.A., Steyermark J.A., Berry P.E., Holst B.K. (1997). Bombacaceae. Flora of the Venezuelan Guayana.

[19] Ducke A. (1930). Plantes nouvelles ou peu connes de la région amazonienne. Arq. Jard. Botânico Rio Jan..

[20] Ducke A. (1937). New forest trees of the Brazilian Amazon. Trop. Wood.

[21] Huber J. (1913). Sobre uma collecção de plantas da região de Cupaty. Bol. Mus. Parana. História Nat. Ethnogr..

[22] Sanoja E. (2004). Diagnosis and observations on the biology of *Catostemma lemense*, new Bombacaceae of Venezuela. Acta Bot. Venez..

